# Synthesis of Elastic Metallic Nickel by Solvent‐Ligand‐Mediated Reductive Assembly

**DOI:** 10.1002/advs.202508160

**Published:** 2025-07-14

**Authors:** Haiyan An, Guoying Tan, Pingru Su, Yu Long, Deyan He, Yu Tang

**Affiliations:** ^1^ State Key Laboratory of Applied Organic Chemistry Key Laboratory of Nonferrous Metal Chemistry and Resources Utilization of Gansu Province College of Chemistry and Chemical Engineering Lanzhou University Lanzhou 730000 P. R. China; ^2^ College of Materials and Energy Lanzhou University Lanzhou 730000 P. R. China

**Keywords:** alkanolamine molecules, electrolysis, large size, metallic nickel, self‐assembly, sponges

## Abstract

Metallic foams hold significant promise as lightweight structural materials. However, most conventional metallic foams are inherently rigid and brittle, frequently undergoing structural collapse. There is an urgent demand to develop cost‐effective, facile‐to‐synthesize metallic foams with enhanced flexibility. Herein, it is discovered that diethanolamine solvent, a multifunctional organic solvent, not only reduces Ni^2+^ ions but also induces the assembly of generated Ni^0^ units into 3D metallic nickel (Ni‐BPHs) sponges. In addition, the optimization of the binary solvent ratio combined with an acetic acid‐assisted method is demonstrated to markedly accelerate the reaction kinetics, concomitant with significantly enhance the coordination density of organic ligands on metallic nickel surface. This approach enables the synthesis of metallic nickel (Ni‐BNSs) sponges with enhanced flexibility. The Ni‐BPHs and Ni‐BNSs as electrocatalysts that exhibit efficient activity and stability for both the hydrogen evolution reaction (HER) and oxygen evolution reaction (OER) in alkaline media. The solvent‐ligand‐mediated reduction and induced assembly strategy has also been demonstrated by extending to other similar ligands. This work marks an important advance in metallic nickel foams and provides a promising strategy for synthesizing mechanically flexible metallic sponges.

## Graphical abstract

The novel elastic metallic nickel, featuring ligands coordinating on the metal surface, is synthesized via rational selection of reduction and structure‐directing organic solvents. Moreover, by modulating solvent composition and incorporating acetic acid ligands, sufficient ligands are enabled to coordinate with the metal surface, thereby improving the mechanical flexibility of metallic nickel, and enabling the fabrication of large‐scale elastic metallic nickel.

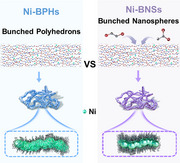

## 1 Introduction

Metallic foams represent a class of 3D resilient nanostructured materials characterized by ultralight weight, low density, and exceptional electrical/thermal conductivity.^[^ ^1^ ^]^ These materials exhibit stochastic porous architectures, wherein the interconnected void spaces substantially enhance material utilization efficiency and overall performance. Their versatile applications span catalysis, fuel cells, hydrogen storage, as well as thermal and acoustic insulation.^[^ ^2^ ^]^ The synthesis of metallic foams primarily employs template‐based strategies.^[^ ^3^ ^]^ For instance, polymer microlattice templates are first fabricated, followed by electroless nickel plating to coat the template surface. Subsequent etching of the template yields ultralight metallic microlattices with hierarchical porosity.^[^ ^4^ ^]^ Alternatively, solution‐phase methods, a conventional approach in the synthesis of metallic foams, typically involve the chemical reduction of metal salts using reducing agents to generate metallic foams.^[^ ^5^ ^]^ Despite their remarkable potential as lightweight structural materials, most existing metallic foams suffer from inherent brittleness and mechanical rigidity, leading to structural collapse under stress, which fundamentally restricts the development of elastic metal.

Organic ligands can serve as structure‐directing agents in soft‐templating approaches.^[^ ^6^ ^]^ Through rational selection of template molecules and optimization of synthesis parameters, mesoporous materials with diverse morphologies and tunable pore sizes have been successfully fabricated.^[^ ^7^ ^]^ However, the framework inevitably undergoes drastic reorganization during calcination for organic template removal or post‐crystallization treatments, often leading to mesostructural shrinkage or even collapse. Additionally, the mesoporous structures formed via soft‐templating are typically amorphous or semicrystalline, which may hinder their practical applications. Tian et al.^[^ ^8^ ^]^ employed F127 as an organic template and utilized an acid‐base pair strategy to modulate inorganic‐inorganic interactions between two or more inorganic precursors. However, the synthesis conditions are highly sensitive to parameters such as solvent purity, temperature, and relative humidity, which critically influence the sol‐gel processes of precursors and thereby disrupt the assembly. In contrast, Zhang et al.^[^ ^6^ ^]^ demonstrated a novel ligand‐assisted assembly strategy, where diblock copolymers acted as structure‐directing agents and acetylacetone functioned as a coordinating ligand. The chelating interactions stabilized hydrolyzed metal nanoparticles, effectively delaying the hydrolysis and condensation of metal precursors and enabling more controllable assembly. Recently, Eychmüller et al.^[^ ^5e^ ^]^ revealed the multifunctional role of inorganic reducing agents (e.g., sodium borohydride, NaBH_4_) as reductant, stabilizer, and initiator during metal gelation. They proposed an excess‐reductant‐directed gelation strategy that integrates ligand chemistry for synthesizing metallic aerogels. Therefore, the development of ligand‐coordination‐assisted methodologies offers a novel strategy for synthesizing stable and crystalline metallic foams. For instance, Pan et al.,^[^ ^9^ ^]^ introduced the small‐molecule‐assisted coordination (e.g., citric acid ligands) during the gelation process, effectively stabilizes metallic gels. Despite significant advancements in synthesis of metallic foams, the development of a universal approach to fabricate stable, highly crystalline metallic sponges remains a formidable challenge. Key limitations include the inherent trade‐offs between structural integrity and crystallinity, as well as the sensitivity of self‐assembly processes to environmental variables.

In this work, we integrated organic ligands as dual‐functional agents, serving both as reducing agents and structural directors in a soft‐templating approach for metal assembly, while leveraging their coordination to enhance material stability. We developed a solvent‐ligand‐mediated strategy that simultaneously achieves the reduction of Ni^2+^ ions and anisotropic assembly of Ni^0^ units into 1D architectures, which further interconnect to form 3D metallic sponges (Ni‐BPHs: metallic nickel bunched polyhedrons). Furthermore, by optimizing the ratio of ethylene glycol (EG) to diethanolamine (DEA) and utilizing acetic acid as co‐ligand, we achieved regulation over the morphology of metallic nickel while ensuring robust coordination of more organic ligands onto the metal surface, yielding metallic nickel sponges with enhanced flexibility (Ni‐BNSs: metallic nickel bunched nanospheres). The two as‐prepared Ni‐BPHs and Ni‐BNSs sponges exhibit exceptional electrocatalytic activity toward both the OER and HER in alkaline media. The solvent‐ligand‐mediated reduction and induced assembly strategy has been demonstrated by extending to other similar ligands, thereby establishing a unique pathway for synthesizing mechanically resilient metallic foams. This work highlights the synergistic interplay between coordination chemistry and nanoscale assembly, provides a promising strategy for engineering advanced porous metallic materials.

## 2 Results and Discussion

Selection of organic ligands: Polyols are now widely used for the preparation of a wide range of inorganic nanoparticles. Interestingly, they can act both as a solvent for solid metal salts and as a reducing agent.^[^ ^10^ ^]^ The multiple OH groups in polyols provide them with the ability to coordinate metal precursors and the reducing properties necessary to reduce electropositive metal ions, such as Ni^2+^ or Co^2+^ ions, to their zero‐valent state, thereby enabling the production of metals in the polyol system. However, nanocrystallite seeds tend to grow into monometallic nanoparticles in the polyol reduction system.^[^ ^11^ ^]^ The appropriate choice of ligand is expected to in situ reduce coordinating metal ions and provide a template‐directed route for the dense parallel assembly of nucleate seed.^[^ ^12^ ^]^ DEA consists of soft donor N atoms and hard donor O atoms, combining the coordinating abilities of nitrogen and oxygen. The soft donor N atoms can bind to relatively softer transition metal ions, while the hard donor O atoms facilitate bridging to metal centers. Additionally, the ligand exhibits tridentate coordination with shorter and more flexible interatomic distances, making it suitable for chelating and bridging metal ions. Consequently, the favored structure can provide a bridging mode with short metal‐metal distances, leading to strong electronic communication.^[^ ^13^ ^]^ Most importantly, DEA contains reductive hydroxyl and amino groups, which is conducive to the in‐situ reduction of coordinating metal ions and the nucleation of metallic clusters. It is well‐established in coordination chemistry that Ni^2+^ ions exhibit strong coordination with N‐ and O‐donor ligands. In light of these considerations, it is imperative that the synthetic protocol simultaneously reduces Ni^2+^ ions through DEA‐mediated electron transfer and induces the anisotropic assembly of Ni^0^ units, to successfully synthesize the intended metallic nickel sponges.

**Scheme 1:**
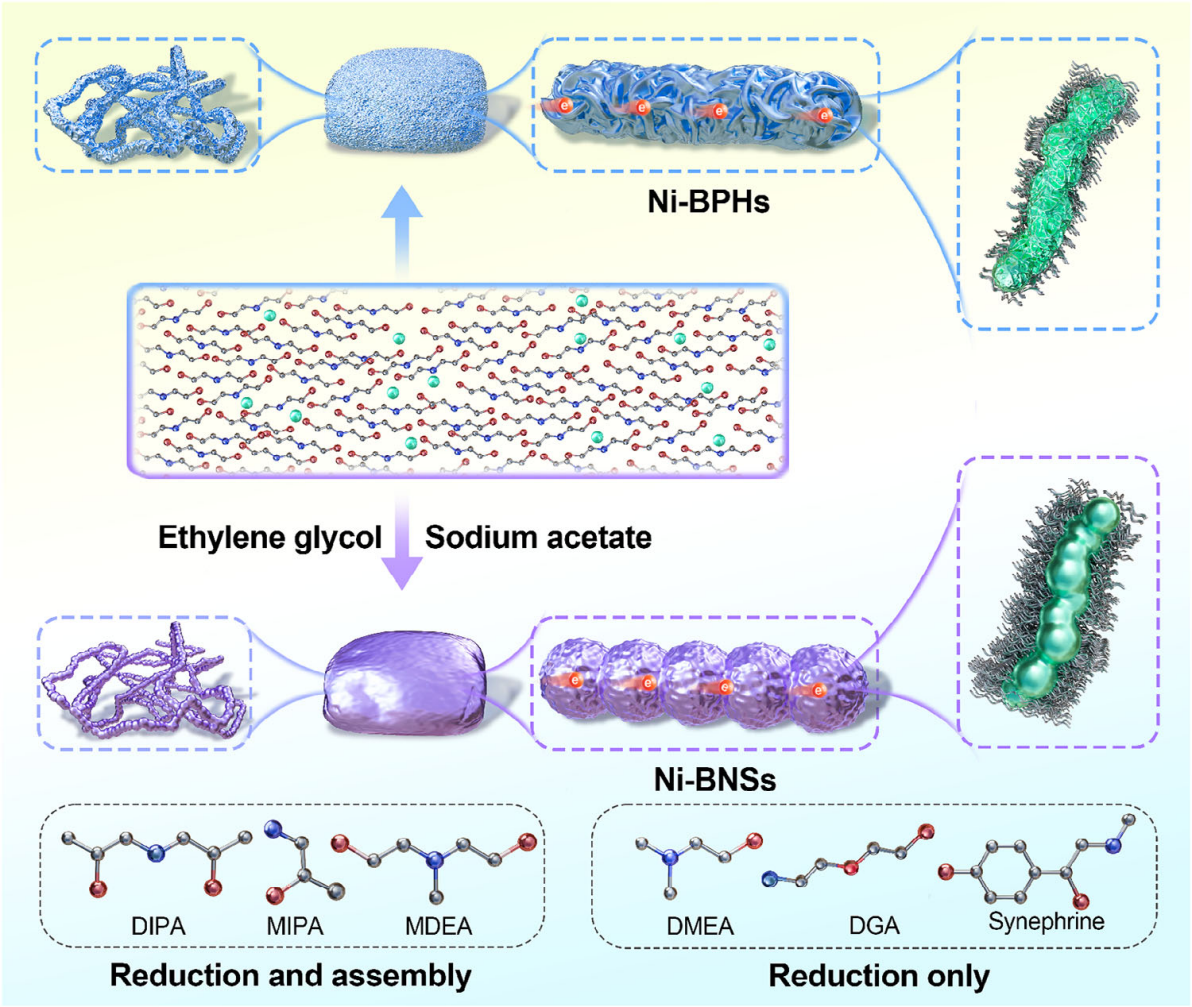
Schematic illustration of the synthesis process for Ni‐BPHs and Ni‐BNSs sponges. Ni‐BPHs sponges were prepared via hydrothermal treatment (200 °C, 10 h) of Ni^2+^ ions with DEA. Ni‐BNSs sponges were synthesized under identical thermal conditions using a mixed solvent system (EG/DEA = 3:1 v/v) with sodium acetate as co‐ligand. Comparison of structurally analogous ligands: Dual‐function ligands (DIPA, MIPA, MDEA) simultaneously reduce Ni^2+^ ions and induce the assembly of Ni^0^ units, reduction‐only ligands (DMEA, DGA, synephrine) exhibit sole Ni^2+^ ions reduction capability without structural guidance.

**Figure 1:**
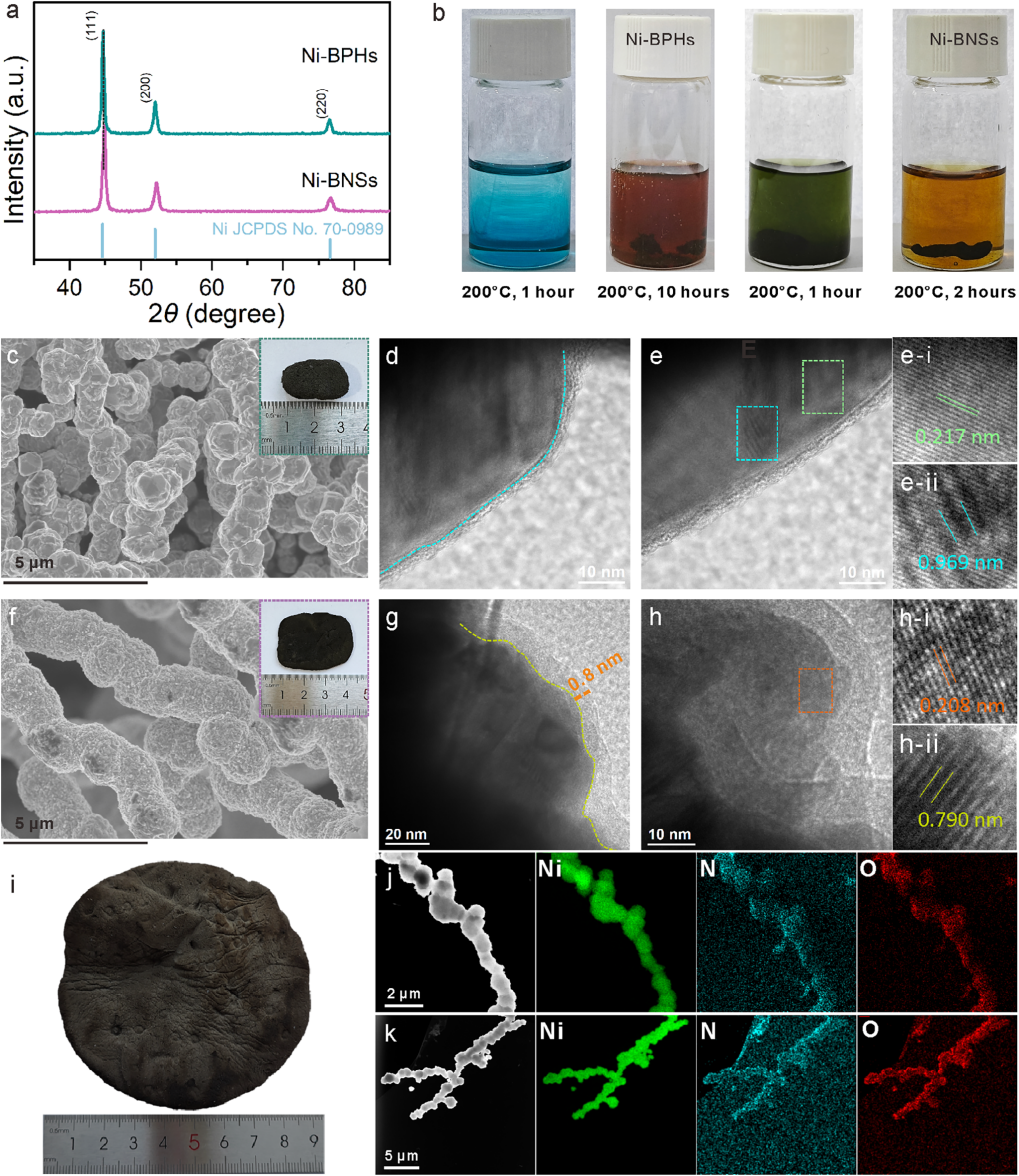
Morphology and structural characterization of the metallic nickel sponges. a) XRD patterns of Ni‐BPHs and Ni‐BNSs. b) Photographs of the reaction solutions of Ni‐BPHs and Ni‐BNSs. c) SEM image of Ni‐BPHs. Insets: Optical image. d,e) TEM images of Ni‐BPHs. e‐i,e‐ii) Corresponding HRTEM images of the areas marked by green and cyan squares in e). f) SEM image of Ni‐BNSs. Insets: Optical image. g,h) TEM images of Ni‐BNSs. h‐i,h‐ii) Corresponding HRTEM images of the areas marked by orange in h) and yellow‐green squares in Figure S12 (Supporting Information). i) Photograph of large‐sized Ni‐BNSs. j) HAADF‐STEM image and corresponding EDS elemental mappings of Ni, N, and O for Ni‐BPHs. k) HAADF‐STEM image and corresponding EDS elemental mappings of Ni, N, and O for Ni‐BNSs.

To highlight the advantage of possible regulation of the synthesis method, two types of samples with distinct surface features were synthesized for comparison. The synthetic route for metallic nickel sponges is illustrated in **Scheme** **1**. The reduction of Ni^2+^ ions to metallic nickel was achieved by employing DEA as both a reducing agent and structural‐directing ligand under solvothermal conditions (200 °C, 10 h), yielding 1D metallic nickel bunched polyhedrons (Ni‐BPHs) (Figure S1, Supporting Information). The bunched architecture originates from anisotropic assembly of polyhedral building blocks, where coordination‐assisted crystallographic orientation drives the formation of polyhedral close‐packed blocks.^[^ ^14^ ^]^ Metallic nickel bunched nanospheres (Ni‐BNSs) assemblies were synthesized via optimizing solvent compositions (EG/DEA = 3:1 v/v) with the controlled addition of sodium acetate (Figure S2, Supporting Information). The X‐ray diffraction (XRD) patterns of Ni‐BPHs and Ni‐BNSs are shown in **Figure** **1**a. The crystalline structure can be assigned to metallic nickel (JCPDS No. 70‐0989), confirming the complete reduction of Ni^2+^ ions and the efficient formation of metallic nickel. This result indicates that the reduction of Ni^2+^ ions can be effectively achieved through the judicious selection of the ligand type. To emphasize the universality of alcohol‐amine molecules, we conducted the following experiment and analysis. Molecular structures similar to that of DEA were preferentially selected as both solvent and reductant. Remarkably, alcohol‐amine molecules such as diisopropanolamine (DIPA), 1‐amino‐2‐propanol (MIPA), N‐methyldiethanolamine (MDEA)) with structures similar to DEA, not only reduced Ni^2+^ ions to generate metallic nickel but also facilitated the fusion of nickel particles, leading to the formation of large‐sized 1D aggregates (Figure S3, Supporting Information). These results verify the effectiveness of alcohol‐amine molecules with similar structures in reduction and structural directing, demonstrating their potential for application in the synthesis of metallic nickel sponges. However, the other alcohol‐amine molecules such as N,N‐dimethylethanolamine (DMEA), diglycolamine (DGA), and synephrine (Figure S4, Supporting Information), primarily function as reducing agents in this system. Under the given conditions, the metallic nickel generated through these reactions typically precipitates as fine powders. After collecting the powder, redispersion was performed in the DEA system. When applying synthetic conditions analogous to the preparation of Ni‐BPHs, DEA failed to induce assembly of pre‐formed metallic nickel particles (Figure S5, Supporting Information). These results demonstrate the importance for conducting in situ reduction and assembly steps in a one‐pot procedure. In control experiments, molecules bearing exclusively either amino group (NH_2_) or hydroxyl group (OH) were reacted with Ni^2+^ ions under controlled conditions. SEM and XRD characterizations (Figure S6, Supporting Information) revealed that these molecules exhibit significantly lower reducing ability compared to alcohol‐amine molecules with both NH₂ and OH groups (e.g., DEA). Furthermore, we investigated the reactivity of other transition metal ions (Fe^2+^, Co^2+^ and Cu^2+^ ions) in the DEA system. Fe^2+^ ions could be not induced to undergo gelation (Figure S7a, Supporting Information). Specifically, the Co^2+^ ions solution remained in its liquid state without precipitation (Figure S7b, Supporting Information). Although Cu^2+^ ions can be reduced by DEA to form metallic Cu aggregates (Figures S7c and S8, Supporting Information), the resulting material appears as a brittle foam.

Representative scanning electron microscopy (SEM) images (Figure 1c,f) reveal that both Ni‐BPHs and Ni‐BNSs exhibit micrometer‐sized bead‐like structures. These structures show a consistent morphological configuration where the bead‐like units are tightly interconnected, forming an extensively entangled 3D network. Notably, Ni‐BPHs displays closely packed arrangement of irregular polyhedra, while Ni‐BNSs showed well‐defined spherical morphology when synthesized in a mixed solvent system with acetic acid as co‐ligand. The gelation of Ni‐BNSs occurred within 2 h at 200 °C, which was significantly faster than that observed in Ni‐BPHs systems (Figure 1b). As also evidenced in prior studies, the introduction of acetic acid serves dual functions: it acts as a chelating agent to modulate the hydrolysis‐condensation kinetics of metal salt while simultaneously regulating the final morphology.^[^ ^15^ ^]^ In addition, the size of the nanospheres showed a concentration‐dependent growth trend, where higher concentration can lead to larger spheres (Figure S9, Supporting Information).

Ni‐BNSs sponges display lower surface roughness compared to Ni‐BPHs sponges (Insets in Figure 1c,f), which correlates with their enhanced mechanical flexibility. Owing to their structural adaptability, Ni‐BNSs can be engineered into various architectures such as cylinders and cuboids (Figure S10, Supporting Information), and further scaled into large‐scale sponges (Figure 1i). Transmission electron microscopy (TEM) analysis revealed distinct morphological features of Ni‐BPHs and Ni‐BNSs. Low‐magnification TEM images (Figure S11, Supporting Information) showed 1D morphology architectures for both materials. High‐resolution TEM (HRTEM) images showed significant differences in edge structures: weak organic layer was observed on the edge structures of Ni‐BPHs (Figure 1d), while an obvious organic layer (∼0.8 nm) was observed on the edge structures of Ni‐BNSs (Figure 1g), potentially arising from enhanced coordination of acetic acid ligands during solvent‐mediated synthesis. The interplanar spacing of 0.217 nm for Ni‐BPHs was assigned to the [111] plane of metallic nickel (Figure 1e‐i),^[^ ^16^ ^]^ and an expanded interlayer distance of ∼0.969 nm was observed in Figure 1e‐ii, which likely corresponds to interlayer spacing matching the (003) crystal plane of Ni(OH)_2_·0.75H_2_O.^[^ ^17^ ^]^ HRTEM image of Ni‐BNSs reveals lattice spacing of 0.208 nm at the edge, corresponding to [111] plane of metallic nickel (Figure 1h‐i). Additionally, the interlayer distance was reduced to ≈0.790 nm (Figure 1h‐ii, the magnified partial view of Figure S12, Supporting Information). This reduced lattice spacing and interlayer distance suggest a more ordered and compact stacking arrangement in the Ni‐BNSs. TEM energy‐dispersive spectroscopy (TEM‐EDS) confirmed homogeneous distribution of Ni, N, and O throughout both architectures (Figure 1j,k).

Fourier‐transform infrared spectroscopy (FT‐IR) was employed to confirm the presence of organic ligands in metallic nickel sponges. The FT‐IR spectra (Figure S13a, Supporting Information) showed characteristic absorption bands at 2850−3025 cm^−1^, assigned to the C–H stretching vibrations of the ─CH_2_ bond.^[^ ^18^ ^]^ Peaks at 1569 and 1646 cm^−1^ were attributed to in‐plane C═N ring stretching coupled with C−H bending modes,^[^ ^19^ ^]^ while the 1045 cm^−1^ peak likely corresponds to C–N stretching vibrations.^[^ ^15^ ^]^ Notably, Ni‐BNSs exhibited stronger band intensities compared to Ni‐BPHs, indicating enhanced stretching vibrations. This observation suggests that acetic acid coordination modifies ligand environment, thereby influencing vibrational intensities. Thermogravimetric analysis (Figure S13b, Supporting Information) revealed total weight losses of 0.79% and 0.42% for Ni‐BNSs and Ni‐BPHs, respectively. The higher ligand content in Ni‐BNSs is attributed to the addition of sodium acetate as a complexing agent during the reaction, which enhances the coordination ability of ligands on the metal surface, increasing the amount of surface‐bound ligands. This is consistent with the TEM results. Complementary FT‐IR/TGA analysis (Figure S13c, Supporting Information) identified 2336 and 2363 cm^−1^ peaks, assigned to the symmetric stretching vibration peak of gaseous CO_2_.^[^ ^20^ ^]^ The convex peaks revealed a clear presence of physisorbed CO_2_,^[^ ^21^ ^]^ in contrast, the concave peaks at the beginning and end indicate a decrease in adsorption. These results confirm the decomposition of organic ligands, thereby underscoring the critical role of organic ligands in stabilizing the metallic nickel phase.

**Figure 2:**
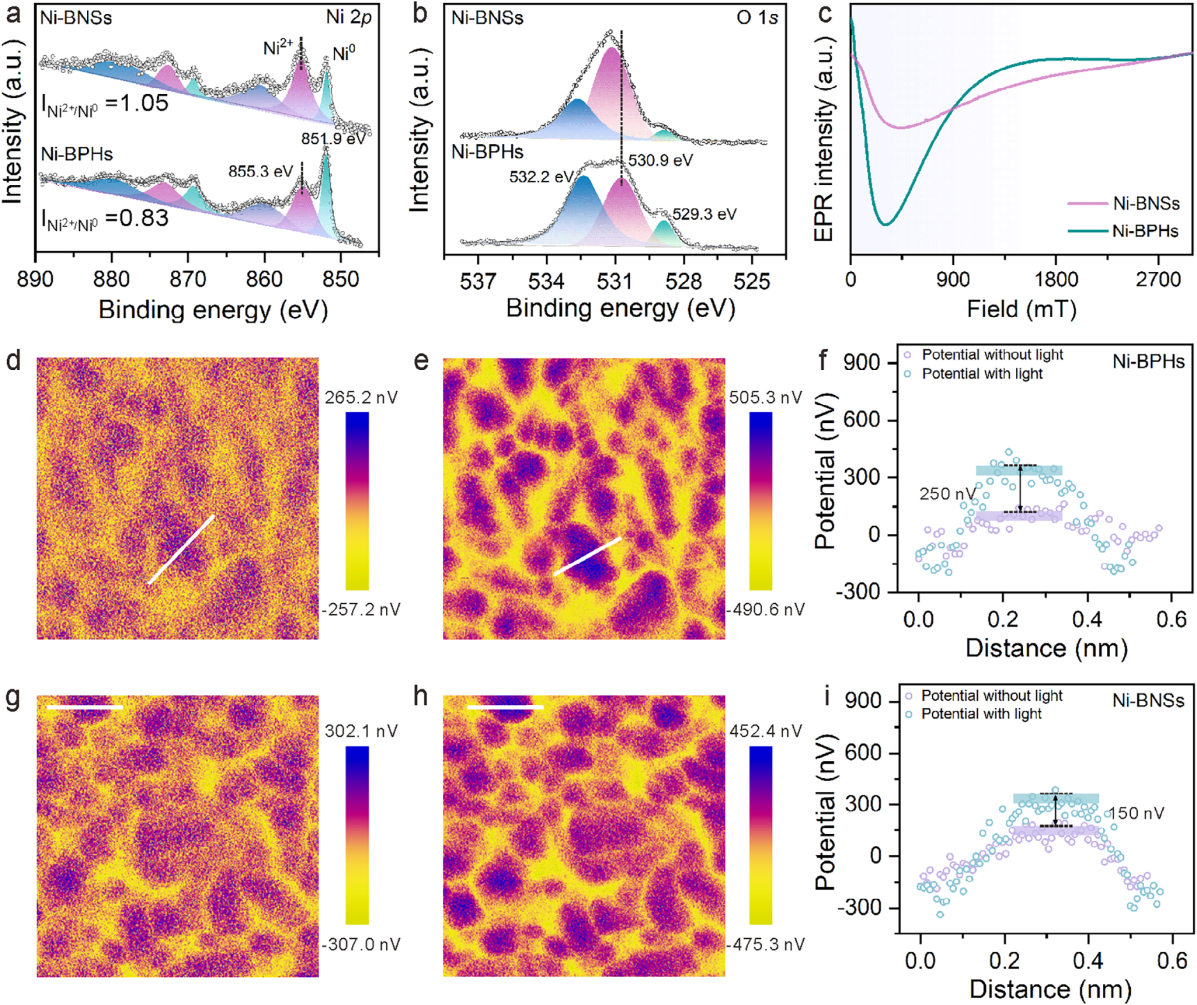
The composition and dipole field characterizations of metallic nickel sponges. a) High‐resolution XPS spectra of Ni 2p for Ni‐BPHs and Ni‐BNSs. b) High‐resolution XPS spectra of O 1s for Ni‐BPHs and Ni‐BNSs. c) EPR spectra of Ni‐BPHs and Ni‐BNSs. d,e) KPFM images of Ni‐BPHs under d) dark and e) light illumination. f) Surface potential profiles derived from KPFM measurements of Ni‐BPHs with and without light. g,h) KPFM images of Ni‐BNSs under g) dark and h) light illumination. i) Surface potential profiles of Ni‐BNSs under dark and light conditions.

The electronic configurations of Ni‐BNSs and Ni‐BPHs were systematically characterized. X‐ray photoelectron spectroscopy (XPS) surface analysis revealed distinct chemical states. The high‐resolution Ni 2*p* spectra of Ni‐BPHs exhibited characteristic peaks at 851.9 eV (metallic Ni^0^) and 855.3 eV (Ni^2+^) (**Figure** **2**a).^[^ ^22^ ^]^ The Ni^2+^ species likely originate from surface oxidation mediated by organic ligands.^[^ ^23^ ^]^ Notably, Ni‐BNSs demonstrated a higher Ni^2+^/Ni^0^ intensity ratio than Ni‐BPHs, attributed to enhanced acetate coordination. The O 1*s* spectra (Figure 2b) exhibited three characteristic peaks at binding energy of 529.3, 530.9 and 532.2 eV, corresponding to Ni─O bonds, surface hydroxides and physisorbed H_2_O, respectively,^[^ ^22^ ^]^ and the peak positions shifted to higher binding energies in Ni‐BNSs compared to Ni‐BPHs. This shift can be attributed to the altered coordination environment induced by acetic acid. In the N 1*s* spectra (Figure S14, Supporting Information), both Ni‐BPHs and Ni‐BNSs displayed characteristic peaks at ∼399.6 eV, which align with the binding energy of secondary amine functional groups.^[^ ^15^, ^24^ ^]^ The presence of oxygen and nitrogen atoms detected by XPS suggests the adsorption of organic molecules on the metallic nickel surfaces, and the observed shift of binding energy and emergence of Ni^2+^ indicate potential interactions between organic molecules and the metal. Electron paramagnetic resonance (EPR) spectra (Figure 2c) showed distinct signals across 0–1800 mT for both Ni‐BNSs and Ni‐BPHs, confirming the existence of unpaired electrons. EPR signals showed a significant intensity enhancement in Ni‐BPHs compared to Ni‐BNSs, indicating higher spin density.

The electronic interactions were further investigated by ultraviolet‐visible diffuse reflectance spectroscopy (UV–vis DRS). As shown in Figure S15 (Supporting Information), the broad absorption spanning 250–600 nm corresponds to ligand‐to‐metal charge transfer transitions and the characteristic d–d transitions of octahedrally coordinated Ni^2+^ species.^[^ ^25^ ^]^ Notably, the redshift is observed in Ni‐BNSs relative to Ni‐BPHs, which may be attributed to the ligand field modulation induced by acetate coordination. Super‐resolution fluorescence microscopy characterization further supports the aforementioned result. Under 487 nm excitation, the fluorescence emission in the blue spectral range was resolved for Ni‐BNSs and Ni‐BPHs (Figure S16, Supporting Information). This spectral signature suggests electronic transitions, this likely arises from strong electrostatic interactions between organic moieties and nickel atoms.^[^ ^26^ ^]^ Kelvin probe force microscopy (KPFM) was employed to characterize the light‐induced surface potential evolution. The contact potential difference (CPD) mappings reveal distinct photoresponse characteristics between Ni‐BPHs, (Figure 2d,e) and Ni‐BNSs (Figure 2g,h) under dark and light conditions. Under light illumination, Ni‐BPHs exhibited an average increase in surface potential of ∼250 nV (Figure 2f), whereas Ni‐BNSs showed a smaller increase in surface potential signal of ∼150 nV (Figure 2i). This pronounced photon‐induced surface potential modulation in Ni‐BPHs and Ni‐BNSs indicates carrier redistribution at the metal‐organic interface.^[^ ^27^ ^]^

**Figure 3:**
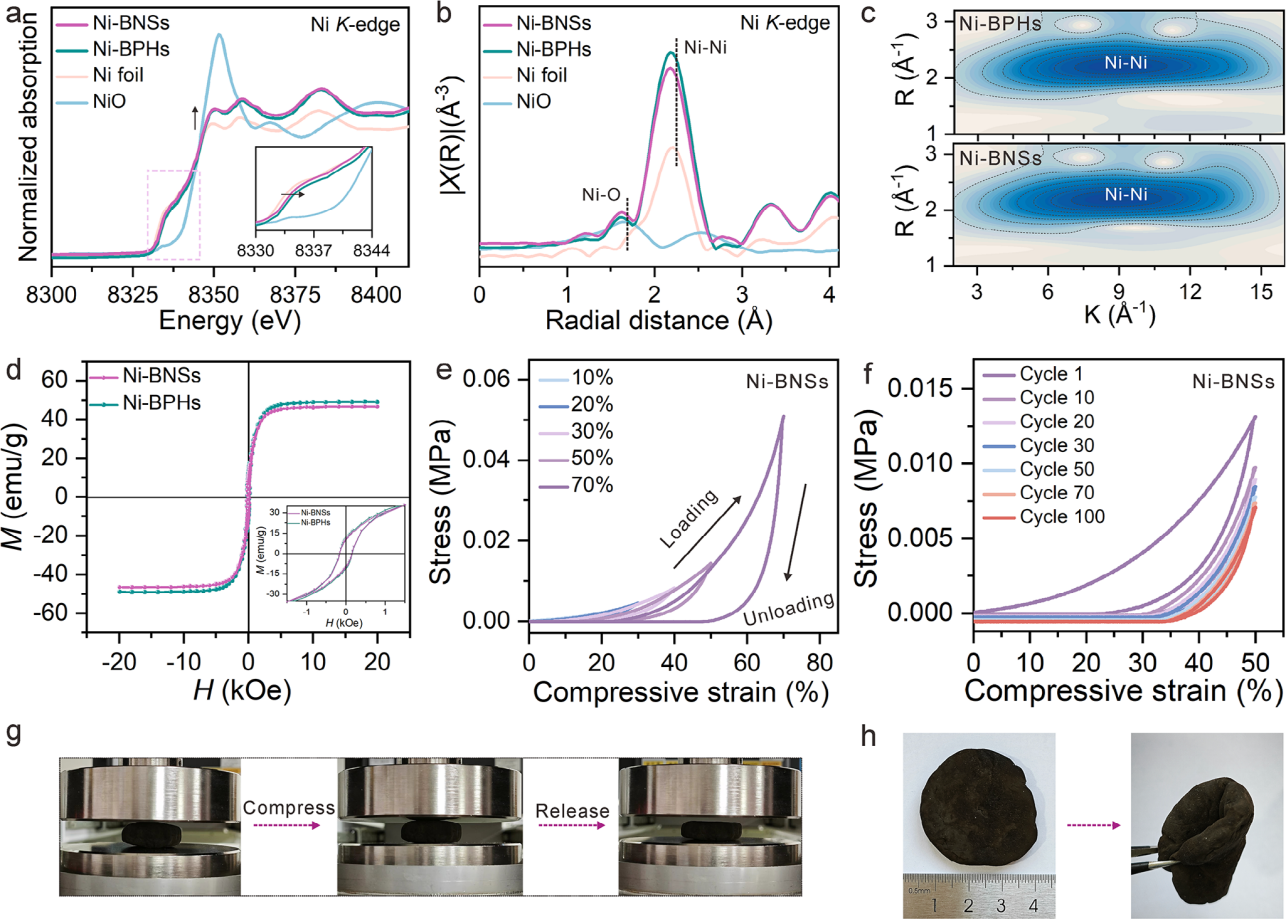
Electronic Structure and stress behavior. a) Normalized Ni *K*‐edge XANES spectra of Ni foil, NiO, Ni‐BPHs, and Ni‐BNSs. The inset in a) shows the partial magnified views of the pre‐edge peak. b) The corresponding *k^3^*‐weighted Fourier transforms of the EXAFS spectra. c) Wavelet transform of *k^3^*‐weighted EXAFS signals for Ni‐BPHs, and Ni‐BNSs. d) Magnetic hysteresis loops for Ni‐BPHs and Ni‐BNSs measured at room temperature. The inset in d) shows an enlargement of the low‐field region. e) Stress‐strain curves during loading‐unloading cycles with increasing strain amplitude. f) A 100‐cycle fatigue test with compressive of 50%. g) Photographs of Ni‐BNSs undergoing compression, illustrating their structural integrity under mechanical stress. h) Photographs showing the flexibility of Ni‐BNSs under bending stress, highlighting their potential for flexible electronic applications.

X‐ray absorption near‐edge structure (XANES) was also employed to investigate the electronic structure and local coordination environment of nickel atom. The normalized Ni *K*‐edge XANES spectra of Ni‐BNSs and Ni‐BPHs (**Figure** **3**a) exhibit features of metallic nickel. Significantly, their absorption edge positions show a slight shift toward higher energy compared to nickel foil, while remaining lower than that of NiO. The intermediate edge position suggests an oxidation state intermediate between Ni^0^ and Ni^2+^,^[^ ^28^ ^]^ confirming the presence of partially oxidized nickel species. Notably, compared to the NiO reference, both Ni‐BNSs and Ni‐BPHs exhibit significantly reduced white‐line intensity that remains slightly higher than that of nickel foil. This spectral feature also demonstrates the coexistence of metallic nickel and oxidized nickel species in Ni‐BNSs and Ni‐BPHs, wherein the metallic phase is dominant as evidenced by the intensity ratio.^[^ ^29^ ^]^ As shown in the Fourier transform (FT) spectra of the *k* ^3^‐weighted Ni *K*‐edge extended X‐ray absorption fine structure (EXAFS) (Figure 3b), the FT‐EXAFS peak position corresponding to metallic Ni─Ni bonds for Ni‐BNSs and Ni‐BPHs appears at a slightly shorter distance (∼2.18 Å) compared to nickel foil (∼2.20 Å). The decreased intensity of the Ni−Ni peak in Ni‐BNSs relative to Ni‐BPHs suggests a reduction in the number of Ni─Ni bonds,^[^ ^30^ ^]^ likely due to the formed Ni─O bonds. Furthermore, the broad peak centered at ∼1.6 Å in the FT spectra corresponds to typical Ni─O bonds,^[^ ^31^ ^]^ which is consistent with the presence of Ni^2+^ species identified by XPS analysis. Additionally, wavelet transform (WT) analysis enhances spatial resolution in both *K*‐space and *R*‐space, enabling precise characterization of the nanoscale coordination environment. As shown in Figure 3c and Figure S17 (Supporting Information), the WT contour plots of Ni‐BNSs and Ni‐BPHs show dominant intensity at R ∼2.1 Å, which is assigned to the Ni─Ni bonds. These results demonstrate the coexistence of both metallic nickel and oxidized nickel species at phase boundaries.

Metals can exhibit magnetic ordering due to aligned electron spins.^[^ ^32^ ^]^ Therefore, the magnetic properties of the Ni‐BNSs and Ni‐BPHs were characterized using vibrating sample magnetometry (VSM) at 300 K. As shown in Figure 3d, both Ni‐BPHs and Ni‐BNSs exhibit typical ferromagnetic hysteresis loops, indicating soft magnetic behavior.^[^ ^33^, ^34^ ^]^ The optical responses of the samples were further characterized using both optical absorption and magnetic circular dichroism (MCD) spectroscopy. The absorption spectra and corresponding MCD spectra of Ni‐BPHs and Ni‐BNSs in ethanol are shown in Figure S18 (Supporting Information). Notably, the distinct feature appearing at ∼238 nm is particularly noteworthy, as this wavelength is associated with the commonly plasmon resonance of metallic nanostructures.^[^ ^35^ ^]^ The minor discrepancies of Ni‐BNSs and Ni‐BPHs in the plasmon resonance spectral positions are likely attributable to different ligand field effects induced by surface‐bound organic molecules.^[^ ^36^ ^]^ Soft magnetic materials generally exhibit good ductility that confer manufacturing flexibility and enable operation.^[^ ^37^ ^]^ To further systematically evaluate the structural flexibility of Ni‐BNSs, we conducted cyclic in‐plane compression tests under controlled strain conditions. Figure 3e displays the stress‐strain curves recorded during five sequential compression cycles with increasing strain amplitudes (10%, 20%, 30%, 50%, 70%). From the compressive stress‐strain curves during loading, linear elastic deformation is observed at strains below 10%, the loading curves show a subsequent plateau stage at 10% to 50%, and a densification regime at 70% strain, where stress increases steeply. From the unloading curves, we can find the permanent residual deformation after each compression cycle, which can be ascribed to the compression of the spaces between microfiber layers.^[^ ^38^ ^]^ To further evaluate the cyclic resilient stability of Ni‐BNSs, compression cycling tests at 50% strain were performed (Figure 3f). The cyclic resilience of the Ni‐BNSs shows a decrease (38% at the 50th cycle and 46% at the 100th cycle) after 100 cyclic compressions, confirming the structural elasticity of Ni‐BNSs. In addition, compared to nickel foam (NF), Ni‐BNSs exhibit superior capability for compressive and bending deformation at low strains (Figure S19a–d, Supporting Information). Figure 3g displays sequential photographs of the compression test. The Ni‐BNSs sponges exhibit limited elastic recovery, as the deformation cannot be fully restored after unloading, which attribute to the decrease in macropore elastic recovery. As shown in Figure 3h and Figure S19e (Supporting Information), the flexural behavior of Ni‐BNSs and their elastic recovery under bending strain loading are demonstrated. This demonstrates that maintaining sufficient ligand coverage on the metal surface can contribute to enhancing metal flexibility and stability.^[^ ^39^ ^]^

**Figure 4:**
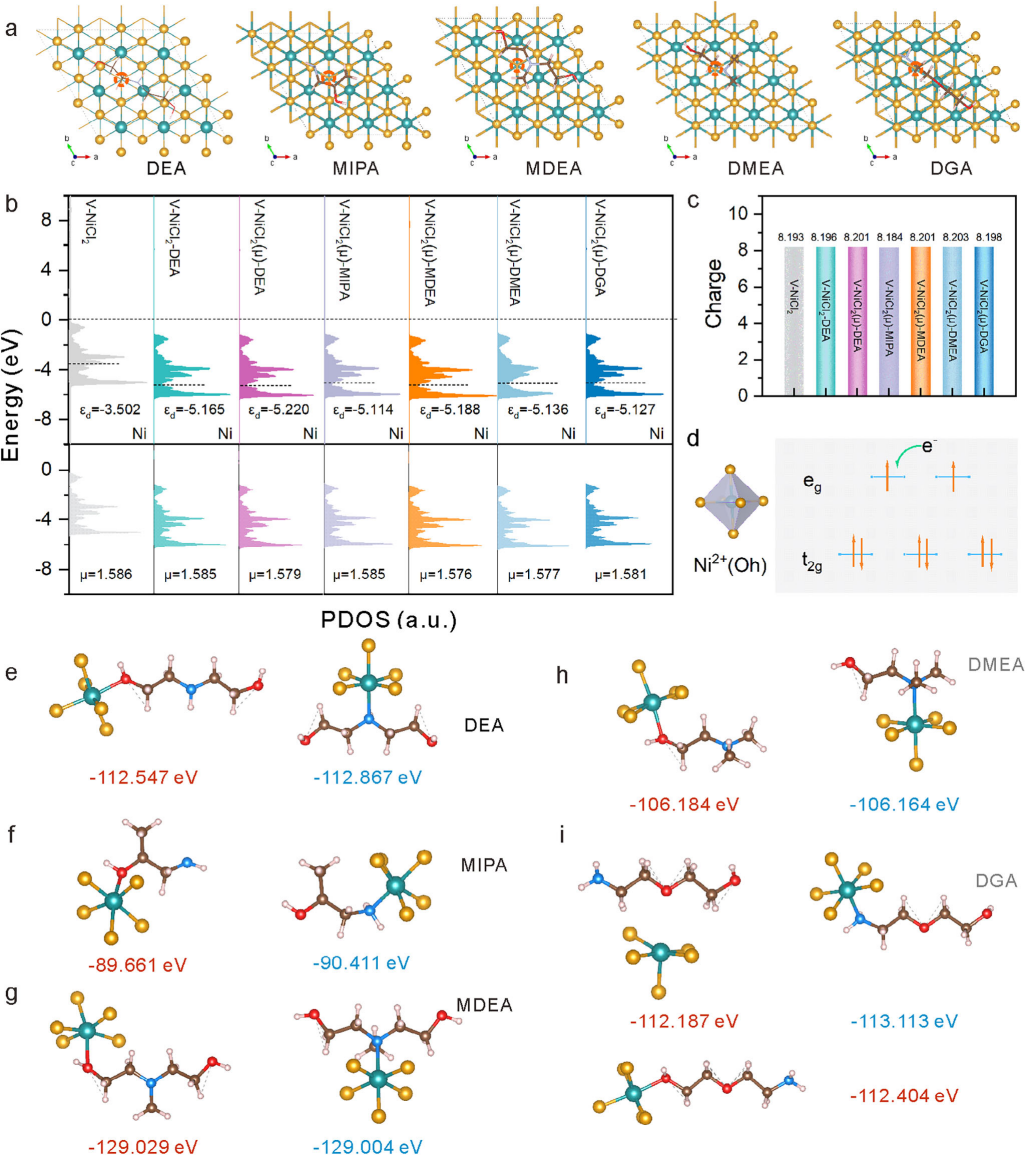
The in‐depth understanding of reduction induced by organic molecular. a) The optimal adsorption structures of DEA, MIPA, MDEA, DMEA, and DGA on the NiCl_2_ crystal structure with a Cl vacancy. b) Calculated PDOS for the d‐band of the Ni species of the V‐NiCl_2_, V‐NiCl_2_‐DEA, V‐NiCl_2_(μ)‐DEA, V‐NiCl_2_(μ)‐MIPA, V‐NiCl_2_(μ)‐MDEA, V‐NiCl_2_(μ)‐DMEA and V‐NiCl_2_(μ)‐DGA. c) Calculated charge of DEA, MIPA, MDEA, DMEA and DGA upon adsorption onto the NiCl_2_ crystal structure. d) Electron arrangement diagram illustrating the electronic interactions between the adsorbates and the NiCl_2_ crystal structure. e‐i) Coordination models of O and N in DEA, MIPA, MDEA, DMEA, and DGA with NiCl_2_, respectively. The coordination energies of O (red) and N (blue) with NiCl_2_ are shown for comparison.

To verify the reduction effect of alcohol‐amine ligands are responsible for the formation of metallic nickel, we systematically investigated reduction of a series of structurally distinct alcohol‐amine ligands. Given that the selected ligands induce stronger ligand fields compared to chloride ligands,^[^ ^36^ ^]^ we expect the replacement of chloride ligands by alcohol‐amine ligands would lead to the formation of nickel coordinated with alcohol‐amine ligands. To probe this, we performed first‐principles calculations to investigate substitution energetics of alcohol‐amine ligands (**Figure** **4**a). The calculated vacancy generation energy for chloride ligand removal (denoted as V‐NiCl_2_) is 2.506 eV (Figure S20, Supporting Information). For example, when DEA coordinates to NiCl_2_ precursor (forming V‐NiCl_2_‐DEA), the vacancy generation energy decreases to 2.504 eV (Figure S21a, Supporting Information). Additionally, the formation of these vacancies is accompanied by the emergence of magnetic moment.^[^ ^40^ ^]^ The V‐NiCl_2_ exhibiting magnetic moment (denoted as V‐NiCl_2_(μ), where μ represents magnetic moment), DEA is coordinated to V‐NiCl_2_(μ) (denoted as V‐NiCl_2_(μ)‐DEA), as shown in Figure S21b (Supporting Information), the vacancy generation energy further decreases to 2.415 eV. These results suggest that DEA facilitates chloride ligand dissociation by lowering the energy barrier of vacancy formation. The emergence of chlorine vacancies correlates with generation of magnetic moment, which further promotes the removal of chloride ligand.

To elucidate the mechanism of DEA‐induced reduction of chloride vacancy formation energy in NiCl_2_, we performed partial density of states (PDOS) analysis on the Ni‐3*d* orbitals. As shown in the top portion of Figure 4b, the Ni *d*‐band center in the V‐NiCl_2_ is located at −3.502 eV relative to the Fermi level. Upon incorporation of DEA (forming V‐NiCl_2_‐DEA), this value shifts downward to −5.165 eV. Remarkably, when accounting for the magnetic moment (V‐NiCl_2_(μ)‐DEA), the Ni *d*‐band center further shifts downward to −5.220 eV, which ultimately lead to a reduction in the chlorine vacancy formation energy. We also note that the antibonding orbitals of the nickel atom in both V‐NiCl_2_‐DEA and V‐NiCl_2_(μ)‐DEA are closer to the Fermi level than those in the V‐NiCl_2_ (Figure S22, Supporting Information), suggesting that these antibonding orbitals of V‐NiCl_2_‐DEA and V‐NiCl_2_(μ)‐DEA are more readily accessible for electron occupation, wherein the increased electron population in antibonding orbitals will weaken the Ni─Cl bond stability, thereby resulting in dissociation of the chloride ligand.

Moreover, the charge associated with *d*‐orbital electrons of the nickel center in V‐NiCl_2_‐DEA and V‐NiCl_2_(μ)‐DEA shows a significant increase compared to that in V‐NiCl_2_ (Figure 4c). This corresponds to a greater electron population at the nickel atom, thereby reducing its oxidation state. Nickel ions in a lower oxidation state are more thermodynamically favorable for further reduction processes, enhancing the dissociation of chlorine ligands. In addition, the increased electron population at the nickel atom suggests that electrons occupy previously singly populated orbitals (Figure 4d), leading to a reduction in the number of unpaired electrons. A decrease in magnetic moment at the nickel center is observed in V‐NiCl_2_‐DEA and V‐NiCl_2_(μ)‐DEA compared to that in V‐NiCl_2_ (the bottom portion of Figure 4b), which further supports the reduction in unpaired electrons. The pairing of electrons in antibonding orbitals directly weakens strength of the Ni─Cl bond by counteracting bonding interactions, thereby promoting the dissociation of chlorine ligands. In parallel, we also investigated alcohol‐amine molecules with structural similarity to DEA, including MIPA, MDEA, DMEA, and DGA, to evaluate their capability in reducing formation energy of chloride vacancy in NiCl_2_. Calculations reveal that compared to pristine V‐NiCl_2_, the Ni *d*‐band center of V‐NiCl_2_(μ)‐MIPA, V‐NiCl_2_(μ)‐MDEA, V‐NiCl_2_(μ)‐DMEA and V‐NiCl_2_(μ)‐DGA (Figure S23a–d, Supporting Information) exhibits a downward shift relative to the Fermi level, a decrease in the magnetic moment at the nickel center (the bottom portion of Figure 4b), and an increase in the charge of d‐orbital electrons at nickel center (Figure 4g; Figure S24, Supporting Information). These observations, combined with XRD analyses (Figures S3a,c and S4a,b, Supporting Information), demonstrate that alcohol‐amine molecules exhibit significant reducing capacity for the reduction of Ni^2+^ to Ni^0^.

The binding energies of N and O atoms in alcohol‐amine ligands coordinated to the Ni center in NiCl_2_ were calculated. As shown in Figure 4e,g,h, the coordination energies of O and N atoms in DEA, MDEA, and DMEA to the Ni center in NiCl_2_ were found to exhibit no significant differences, which implied that both N and O atoms could act as competitive coordination sites. Compared to O atoms, N atoms at terminal positions in MIPA (Figure 4f) and DGA (Figure 4i) exhibited markedly lower binding energies (N: −90.411 eV, −113.113 eV *vs*. O: −89.661 eV, −112.404 eV), which suggests a thermodynamic preference for N‐dominated coordination. These analyses indicate that the potential formation of stable metal–ligand bonds in the reaction. It is noteworthy that chain‐structured molecules such as MIPA and MDEA, which display structural similarities with DEA in both chain conformation and spatial arrangement of N and O atoms, can induce the assembly of generated Ni^0^ units into chain skeletons (see Figures S3a,c, Supporting Information). However, when the chain conformation of DMEA shows differences compared to DEA, the desired chain structure cannot be fabricated (see Figure S4a, Supporting Information). Generally, upon reduction of Ni^2+^ by organic molecules, the dispersion‐aggregation behavior of the resulting metallic nickel is fundamentally governed by organic molecule coordination at the metal surface. This behavior is inherently linked to the their specific coordination mode and chemical properties of the organic molecules.^[^ ^41^ ^]^ Thus, the formation of linear chains through the self‐assembly of nickel nanoparticles coordinated with organic molecules is mainly attributed to the steric hindrance from surface‐bound molecules architecture and long‐range electrostatic repulsions,^[^ ^42^ ^]^ wherein the molecular architecture is predominantly governed by bond directionality, which dictates the spatial arrangement and structural torsion.^[^ ^43^ ^]^ Therefore, the self‐assembly of metallic nickel in the synthesis of Ni‐BPHs and Ni‐BNSs is mainly driven by synergistic metallic bonding and supramolecular interactions.^[^ ^44^ ^]^

**Figure 5:**
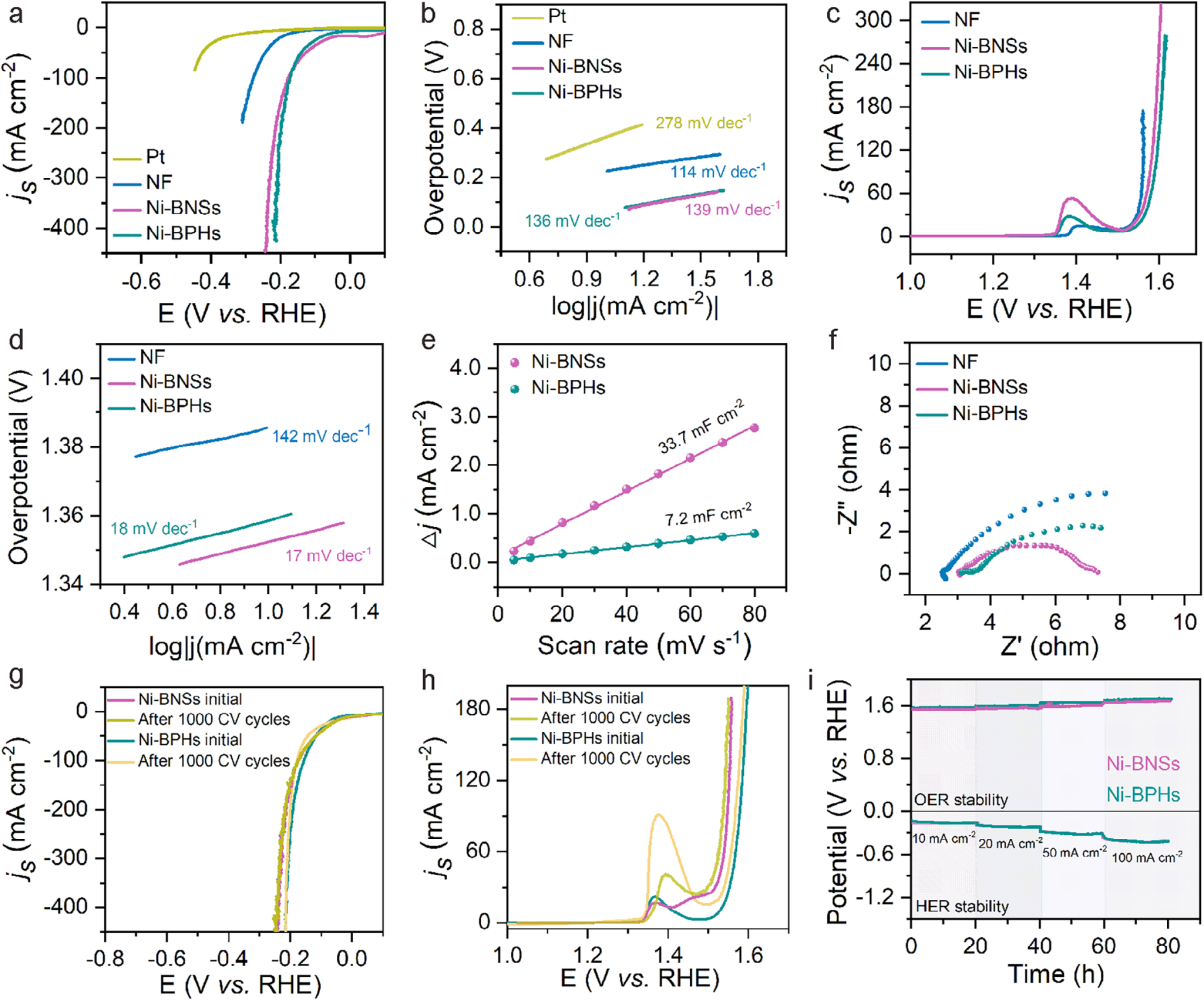
HER and OER performance of metallic nickel sponges. a) LSV polarization curves for the HER and b) the corresponding Tafel plots of Pt, commercial NF, Ni‐BPHs, and Ni‐BNSs in 1.0 M KOH. c) LSV polarization curves for the OER and d) the corresponding Tafel plots of NF, Ni‐BPHs, and Ni‐BNSs. e) Linear fitting of the current density versus scan rates for Ni‐BPHs, and Ni‐BNSs, and the corresponding C_dl_ values. f) EIS Nyquist curves for Ni‐BPHs and Ni‐BNSs. g) LSV polarization curves for the HER of Ni‐BPHs and Ni‐BNSs before and after 1000 cyclic voltammetry cycles. h) LSV polarization curves for the OER of Ni‐BPHs and Ni‐BNSs before and after 1000 cyclic voltammetry cycles. i) long‐term stability tests of the Ni‐BPHs and Ni‐BNSs at different current densities: 10; 20; 50; and 100 mA cm^−2^.

Magnetic transition metals exhibit superior catalytic performance due to their unique spin‐polarized electronic configurations, particularly exhibiting exceptional potential in sustainable energy conversion processes such as hydrogen evolution and oxygen evolution reactions.^[^ ^9^, ^45^ ^]^ To explore potential electrocatalytic activity of Ni‐BPHs and Ni‐BNSs sponges, we first evaluated their HER activity. **Figure** **5**a presents the iR‐corrected linear sweep voltammetry (LSV) curves of Ni‐BPHs, Ni‐BNSs, Pt and NF in 1 m KOH. Compared to Pt (overpotential η = 364 mV) and NF (η = 202 mV), the Ni‐BNSs and Ni‐BPHs sponges exhibit significantly enhanced HER activity, requiring overpotentials of only 56 and 88 mV, respectively, to achieve a current density of 20 mA cm^−2^. The Tafel slope of Ni‐BPHs (136 mV dec dec^−1^) is essentially comparable to that of Ni‐BNSs (139 mV dec dec^−1^) (Figure 5b), suggesting that both materials follow similar rate‐determining steps in the HER process. The electrochemically active surface area (ECSA), a critical parameter for evaluating catalytic active sites, was determined by calculating the electrochemical double‐layer capacitance (C_dl_) from cyclic voltammetry (CV) measurements (Figure S25, Supporting Information). Ni‐BNSs show a C_dl_ value of 11.8 mF cm^−2^, which is markedly higher than that of Ni‐BPHs (1.9 mF cm^−2^) (Figure S26, Supporting Information). The increased C_dl_ suggests that Ni‐BNSs exposes a larger number of catalytically active sites compared to Ni‐BPHs. Electrochemical impedance spectroscopy (EIS) revealed that Ni‐BNSs exhibited a significantly lower charge transfer resistance compared to Ni‐BPHs, as evidenced by the smaller semicircle diameter in Nyquist plots (Figure S27, Supporting Information). For the OER, NF exhibits a low overpotential of 307 mV at 20 mA cm^−2^ in alkaline media. In comparison, Ni‐BNSs and Ni‐BPHs exhibit competitive performance with overpotentials of 313 and 325 mV, respectively, at the same current density (Figure 5c). The Tafel slopes of Ni‐BNSs and Ni‐BPHs are as low as 17 and 18 mV dec^−1^ (Figure 5d), further confirming their favorable reaction kinetics. Additionally, the C_dl_ of Ni‐BNSs reaches 33.7 mF cm^−2^, which is significantly higher than that of Ni‐BPHs (7.2 mF cm^−2^) (Figure 5e; Figure S28, Supporting Information). This difference highlights the advantage of the morphology in exposing abundant catalytic active sites. EIS further corroborates the superior charge transfer capability of Ni‐BNSs and Ni‐BPHs compared to NF (Figure 5f). The specific surface area of Ni‐BPHs shows marginally enhanced specific surface area over Ni‐BNSs (Figure S29, Supporting Information), however, ultralow specific surface area is statistically insignificant. Thus, the difference in specific surface area may be caused by errors. The observed exceptionally low specific surface area is likely attributed to the solid, micron‐sized structures, which predominantly expose exterior surfaces contributing to the measurable specific surface area. Consequently, a substantial number of active sites remain inaccessible, thereby constraining the electrocatalytic performance.

The stability of Ni‐BNSs and Ni‐BPHs was systematically evaluated through the continuous CV scans and chronopotentiometry. CV stability tests were conducted at a scan rate of 50 mV s^−1^ for 1000 cycles. After this prolonged cycling, the HER overpotential of Ni‐BNSs at 20 mA cm^−2^ almost unchanged, while Ni‐BPHs exhibited a degradation with a 10 mV (Figure 5g). The OER overpotential of Ni‐BNSs and Ni‐BPHs at 20 mA cm^−2^ exhibit minimal activity increase (Figure 5h). Although the oxidation peak shifts to higher potentials after repeated cycling, which implies that the electrochemical oxidation is thermodynamically less favorable,^[^ ^46^ ^]^ both Ni‐BNSs and Ni‐BPHs exhibit a significantly enhanced oxidation peak. This is attributed to the formation of low‐crystallinity species, which significantly promotes the exposure of OER active sites and thereby exhibiting higher electrocatalytic activity.^[^ ^47^ ^]^

The long‐term stability of Ni‐BNSs and Ni‐BPHs under practical operating conditions was systematically evaluated through chronopotentiometric measurements at current densities of 10, 20, 50, and 100 mA cm^−2^ over 20 h (Figure 5i). For HER, Ni‐BNSs exhibited overpotential increases of 38, 28, 36, and 27 mV at 10, 20, 50, and 100 mA cm^−2^, respectively. In comparison, Ni‐BPHs showed increases of 29, 17, 25, and 26 mV under the same conditions. For OER, after 20 h of operation, Ni‐BNSs demonstrated overpotential increments of 3, 2, 11, and 24 mV at the respective current densities. However, Ni‐BPHs showed a mixed trend with increases of 12, 11, 4, and 21 mV. The stability tests reveal that both Ni‐BNSs and Ni‐BPHs maintain catalytic durability, showing minimal activity decay in electrocatalytic process. Their nickel contents detected by inductively coupled plasma‐mass spectrometry (ICP‐MS) (Tables S1, Supporting Information), demonstrating organic molecules act as a barrier layer to slow down the dissolution of Ni‐BNSs and Ni‐BPHs. The observed performance decay is likely attributed to dynamic chemical transformation of organic ligands and/or active site obstruction by gas bubbles during prolonged electrocatalysis, thereby collectively deteriorating the catalytic activity. Impressively, both Ni‐BNSs and Ni‐BPHs exhibit competitive performance compared with the other reported Ni‐based electrocatalysts in alkaline media (Tables S2 and S3, Supporting Information).

To identify the catalytically active species under operational conditions, we conducted post‐electrolysis characterizations. The XPS analysis of Ni‐BNSs and Ni‐BPHs after the electrochemical reaction reveals significant surface evolution. In the Ni 2*p* spectra of Ni‐BNSs (Figure S30a, Supporting Information), the characteristic Ni^0^ peak at 851.9 eV disappears, accompanied by a slight positive shift of the Ni^2+^ peak, indicating enhanced oxidation states. Concurrently, the O 1*s* signal associated with surface hydroxides shifts to lower binding energy (Figure S30b, Supporting Information), suggesting strengthened Ni–O interactions during surface structure reorganization. Notably, the attenuation of the N 1*s* peak at 399.7 eV (assigned to N─Ni─O bonding^[^ ^48^ ^]^) (Figure S30c, Supporting Information) implies partial dissociation of coordinated alkanolamine molecules. Similar trends are observed for Ni‐BPHs (Figure S30g‐i, Supporting Information). These analyses corroborate the electrochemically driven surface oxidation, demonstrating a dynamic reconstruction process under operating conditions.

The XRD pattern of Ni‐BNSs and Ni‐BPHs after HER (Figure S31, Supporting Information) shows characteristic peaks corresponding to the metallic nickel phase. Notably, no other phases (e.g., nickel oxides or hydroxides) are detected. However, the XRD pattern of Ni‐BNSs and Ni‐BPHs retains the characteristic diffraction peaks of metallic nickel phase, and new distinct peaks emerge at ∼32° after OER. This result demonstrates that the surface of Ni‐BNSs and Ni‐BPHs undergo structural reorganization under OER conditions, forming a new phase identified as the active species for the OER. To elucidate the reconstruction dynamics, we conducted in situ Raman spectroscopy for monitoring the potential‐dependent structural evolution of Ni‐BNSs and Ni‐BPHs under applied potential. As shown in Figure S32 (Supporting Information), two peaks at ≈475 and 557 cm^−1^ emerged beyond 1.4 V, corresponding to the E_g_ bending (Ni─O─Ni bending) and A_1g_ stretching (O─NivO stretching) vibrations of Ni^3+^–O in γ‐NiOOH, respectively.^[^ ^25^ ^]^ This indicates that the formed γ‐NiOOH is surface reconstruction species of Ni‐BNSs and Ni‐BPHs during OER, which serves as the catalytically active species. The characteristic peaks of γ‐NiOOH remained nearly constant with increasing voltage, suggesting that deep surface restructuring of Ni‐BNSs and Ni‐BPHs was suppressed.^[^ ^49^ ^]^ The in situ oxidative reconstruction of the pristine Ni‐ligand phase into γ‐NiOOH induces robust interfacial coupling effects, thereby modulating the electronic configuration of nickel active sites, reducing adsorption energy barriers, and enhancing charge transfer from active sites to the substrate. This synergistic process collectively enables exceptional catalytic performance.^[^ ^50^ ^]^

## 3 Conclusion

In summary, this work has successfully tackled the intrinsic brittleness and rigidity of metallic foams by developing a inspiring strategy that utilizes organic solvents for simultaneous reduction and induction assembly. Specifically, the organic solvent not only reduces Ni^2+^ ions to metallic Ni^0^ but also mediates the assembly of generated Ni^0^ units into 3D structure. In addition, the control over the morphology of the as‐synthesized metallic nickel was achieved by modulating the solvent compositions and employing an acetic acid ligand‐assisted approach, which also simultaneously enhanced assembly efficiency of metallic nickel and enabled sufficient ligands to coordinate on the metal surface, resulting in a metallic sponge with enhanced mechanical flexibility and structural resilience. The as‐synthesized Ni‐BPHs and Ni‐BNSs sponges exhibit exceptional electrocatalytic performance for both the HER and OER in alkaline media. The cost‐effectiveness of the organic solvents and nickel salt positions the metallic nickel sponges as a viable candidate for large‐scale applications across diverse fields in the future. Furthermore, the solvent‐mediated reduction and assembly strategy can be extended to other metal‐ligand systems, which paves the way for synthesizing mechanically robust and multifunctional metallic sponges.

## Conflict of Interest

The authors declare no conflict of interest.

## Supporting information



Supporting Information

## Data Availability

Research data are not shared.
