# Supplementary material for: Synthesis of Elastic Metallic Nickel by Solvent‐Ligand‐Mediated Reductive Assembly
Source: Adv Sci (Weinh). 2025 Jul 14;12(38):e08160. doi: 10.1002/advs.202508160 (PMC12520517; doi:10.1002/advs.202508160)
Supplement: Supplementary file 1 — Supporting Information [file ADVS-12-e08160-s001.docx]

Supporting Information

**Synthesis of Elastic Metallic Nickel by Solvent-Ligand-Mediated Reductive Assembly**

*Haiyan An^†^, Guoying Tan^†^, Pingru Su, Yu Long, Deyan He*, and Yu Tang**

H. An, G. Tan, P. Su, Y. Long, Y. Tang

State Key Laboratory of Applied Organic Chemistry, Key Laboratory of Nonferrous Metal Chemistry and Resources Utilization of Gansu Province, College of Chemistry and Chemical Engineering, Lanzhou University, Lanzhou 730000, P.R. China

E-mail: tangyu@lzu.edu.cn

H. An, D. He

College of Materials and Energy, Lanzhou University, Lanzhou 730000, P.R. China

E-mail: hedy@lzu.edu.cn

^†^These authors contributed equally to this work.

Materials and Methods

**Chemicals**

The chemical reagents used are as follows: Nickel(II) chloride hexahydrate (NiCl_2_·6H_2_O), sodium acetate anhydrous (NaAc). diethanolamine (DEA), 1-amino-2-propanol (MIPA), diglycolamine (DGA), diisopropanolamine (DIPA), synephrine, 1,2-diaminobenzene, benzamide, melamine, ethylenediamine, triethylamine, *N,N*-dimethylethanolamine (DMEA), *N*-methyldiethanolamine (MDEA). Iron(III) chloride hexahydrate (FeCl_3_·6H_2_O), Cobalt(II) chloride hexahydrate (CoCl_2_·6H_2_O), copper(II) chloride dihydrate (CuCl_2_·2H_2_O). Ethylene glycol (EG) and ethanol (C_2_H_5_OH, AR) were obtained from commercial suppliers and used without further purification.

**Synthesis of Ni-BPHs sponges**

NiCl_2_·6H_2_O (1 mmol, 237.7 mg) was directly added into DEA (20 mL). The solution was then transferred to a 50 mL Teflon-lined stainless-steel autoclave and kept in an oven at 200 ^o^C for 10 h. The reaction vessel was cooled to room temperature naturally, and the product was washed several times with ethanol, then dried overnight.

**Synthesis of Ni-BNSs sponges**

NiCl_2_·6H_2_O (1 mmol, 237.7 mg) was directly added into EG (15 mL). After the NiCl_2_·6H_2_O dissolved, and then DEA (5 mL) and NaAc (9 mmol, 738.3 mg) were successively added into EG solution under magnetic stirring. The reaction mixture was stirred for 30 min at room temperature. The precursor was then transferred to a 50 mL Teflon-lined stainless-steel autoclave and kept in an oven at 200 ^o^C for 10 h. The reaction vessel was cooled to room temperature naturally, and the product was washed several times with ethanol, then dried overnight.

**Preparation of other Ni-based materials**

The preparation process was same as that of Ni-BPHs, except that DEA was replaced by 1-amino-2-propanol, diglycolamine, diisopropanolamine, synephrine, trolamine, 1,2-diaminobenzene, benzamide, melamine, ethylenediamine, triethylamine, *N,N*-dimethylethanolamine, *N*-methyldiethanolamine, respectively.

**Preparation of other Fe, Co, Cu-based materials**

The preparation process was same as that of Ni-BPHs, except that NiCl_2_·6H_2_O was replaced by FeCl_3_·6H_2_O, CoCl_2_·6H_2_O, CuCl_2_·2H_2_O, respectively.

Instrumentation

The Powder X-ray diffraction (XRD), which was conducted on a Rigaku MiniFlex600 X-ray diffractometer using CuKa radiation. Scanning electron microscopy (SEM) analysis was carried out on a Zeiss Gemini 300 field emission scanning electron microscope. The surface images were recorded on a transmission electron microscopy (TEM, talos F200s) at 200 kV and 100 K. X-ray photoelectron spectra (XPS) spectra were recorded on a Shimadzu Axis Supra device, and the binding energy was calibrated relative to the signal of C 1*s* peak at 284.8 eV. Raman spectra of samples were measured with a Lab RAM HR Evolution (532 nm). The Fourier-transform infrared spectroscopy (FT-IR) were recorded by using Nicolet iS50 spectrometer with KBr pellets technique. Thermogravimetric analyses (TGA) were performed on a Mettler Toledo Star System under a nitrogen atmosphere at a heating rate of 10 °C·min^−1^. UV-vis absorption and UV-vis diffuse reflection spectra (UV–vis DRS) were obtained on UV-2600 (BaSO_4_ as reference). Electron paramagnetic resonance (EPR) spectra were performed on electron paramagnetic resonance spectrometer (ER200DSRC10/12). Inductively coupled plasma-mass spectrometry (ICP-MS) was carried out to determine the element contents of the samples. The Kelvin probe force microscopy (KPFM) of the samples were measured by atomic scanning probe microscope and surface photovoltage spectrum using a Dimension-Icon (Bruker). X-ray absorption spectroscopy (XAS) data were obtained from xueyanhui testing institution. Data were processed according to standard procedures using the Demeter program package.

Electrochemical measurements

The Electrochemical measurements were conducted in a typical three-electrode glass cell by an Electrochemical Workstation (CHI 760E). A saturated Hg/HgO electrode and Pt plate were used as reference electrode and counter electrode, respectively, the working electrode is pressed and cut metallic nickel sponges (1×0.5 cm^2^). The potential was converted to reversible hydrogen electrode (RHE) via a Nernst equation: E (RHE) = E (Hg/HgO) + 0.059 × pH + 0.098 V. The overpotential (η) was calculated by η (V) = E (RHE) - 1.23 V. Cyclic voltammetry (CV) and Linear sweep voltammetry (LSV) measurements were recorded at a scan rate of 5 mV s^-1^ in 1 M KOH solution. All polarization curves were calibrated with iR correction. Electrochemical impedance spectroscopy (EIS) was measured with a frequency range from 100 KHz to 0.01 Hz. The electrochemical double-layer capacitance (C_dl_) was measured by CV at a potential window using the same working electrodes. CV curves were obtained at different scan rates of 5, 10, 20, 30, 40, 50, 60,70, 80 mV s^-1^. The current differences against scan rates were fitted to analyze electrochemically active surface area (ECSA).

Theoretical calculation

The Density functional theory (DFT) calculations are performed by using the Vienna Ab-initio Simulation Package (VASP).^[1]^ Projector augmented wave (PAW) method^[2]^ was used. The exchange and correlation energy density function were described by the Generalized Gradient Approximation (GGA) of Perdew-Burke-Ernzerh (PBE).^[3]^ To describe the correlation for 3*d* transition metal systems, the DFT+U method was used. The value of U was 3.40 for Ni. The cutoff energy for plane-wave basis function was chosen to be 450 eV. The maximum energy and force change of 10^−4^ eV and 0.02 eV/Å respectively. The 3×3×1 Monkhorst-Pack type of K-point set up was used for all surface structural models. Choose (001) surface of NiCl_2_ and the vacuum layer set to 30 Å. All the surfaces were modeled by a periodically repeated 3 × 3 unit cell in the x- and y-directions.


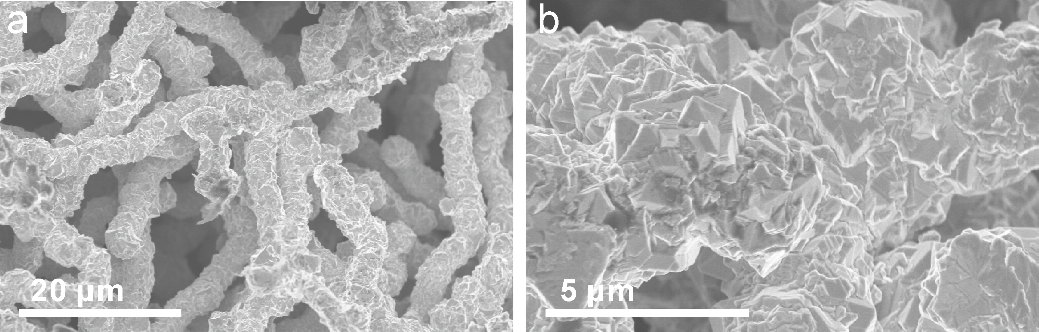


**Figure S1.** SEM images of Ni-BPHs sponges.

**
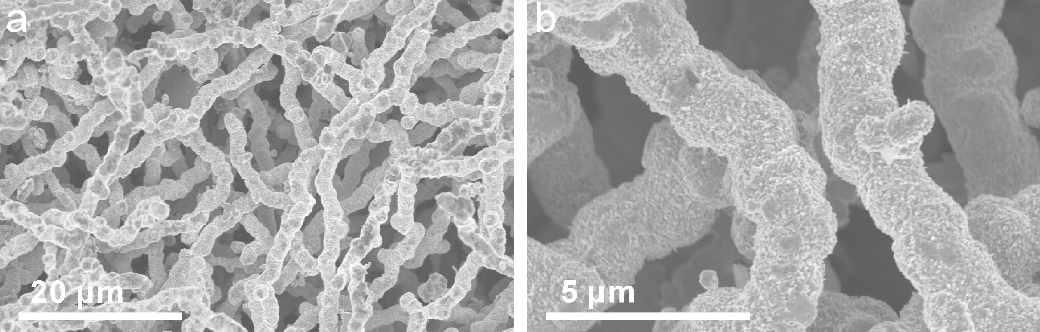
**

**Figure S2.** SEM images of Ni-BNSs sponges.

The following preparation process was same as that of Ni-BNSs, except that the volume ratio of EG to DEA has been adjusted. (1) The volumes of EG and DEA were adjusted to 10 mL and 10 mL, respectively. The obtained product is powdery metallic nickel, its macroscopic state is a linear dispersion rather than spongy metallic nickel. (2) The volumes of EG and DEA were adjusted to 16 mL and 4 mL, respectively. The obtained product is metallic nickel sponges. (3) The volumes of EG and DEA were adjusted to 19 mL and 1 mL, respectively. The macroscopic state of obtained product is a linear dispersion rather than spongy metallic nickel, and the yield is also significantly low.

Comparative experiments of procedures (1), (2), and (3) demonstrate that the EG-to-DEA volume ratio governs metallic nickel sponge formation. Consequently, we selected EG and DEA volumes of 15 mL and 5 mL, respectively.

The following preparation process is same as that of Ni-BPHs, except that the dosage of NaAc has been adjusted. (1) The NaAc was was not added. After the reaction, the reaction solution was clear, and no product was formed. (2) The dosage of NaAc was adjusted to 5 mmol, and the obtained product was metallic nickel sponges. (3) The dosage of NaAc was adjusted to 15 mmol, and the obtained product was metallic nickel sponges. The macroscopic state of obtained product is a linear dispersion rather than spongy metallic nickel.

Comparative experiments of procedures (1), (2), and (3) demonstrate that the NaAc also governs metallic nickel sponge formation. Therefore, we chose to add 9 mmol of NaAc to produce a higher yield of metallic nickel sponges.

Furthermore, we modulated reaction temperatures to 160°C, 170°C, and 180°C. At 160°C (10 h reaction), the product manifested as linear dispersoids macroscopically. The 170°C reaction (10 h) yielded structurally inferior assemblies lacking coherence. Reaction at 180°C (10 h) produced a consolidated monolithic structure. Meanwhile, we also investigated that in an EG solution of Ni^2+^ ions, the addition of only the NaAc (without DEA) failed to produce spongy metallic nickel.

Based on the above adjustment of reaction conditions, we selected 15 mL EG and 5 mL DEA (EG:DEA ratio 3:1) with 9 mmol of NaAc, conducting the reaction at 200°C to obtain a better metallic nickel sponges.

Therefore, Regarding the influence of solvent composition on the self-assembly process, we think that DEA features two hydroxyl groups and an additional amino group, thereby imparting enhanced reducing capacity and more extensive intermolecular hydrogen bonding, which permits direct reaction with nickel salts and promote gelation. EG contains two hydroxyl groups, exhibiting lower viscosity and reducibility. Therefore, the addition of DEA is required to enhance reducing capacity, while NaAc must be incorporated as a complexing agent to facilitate gelation.


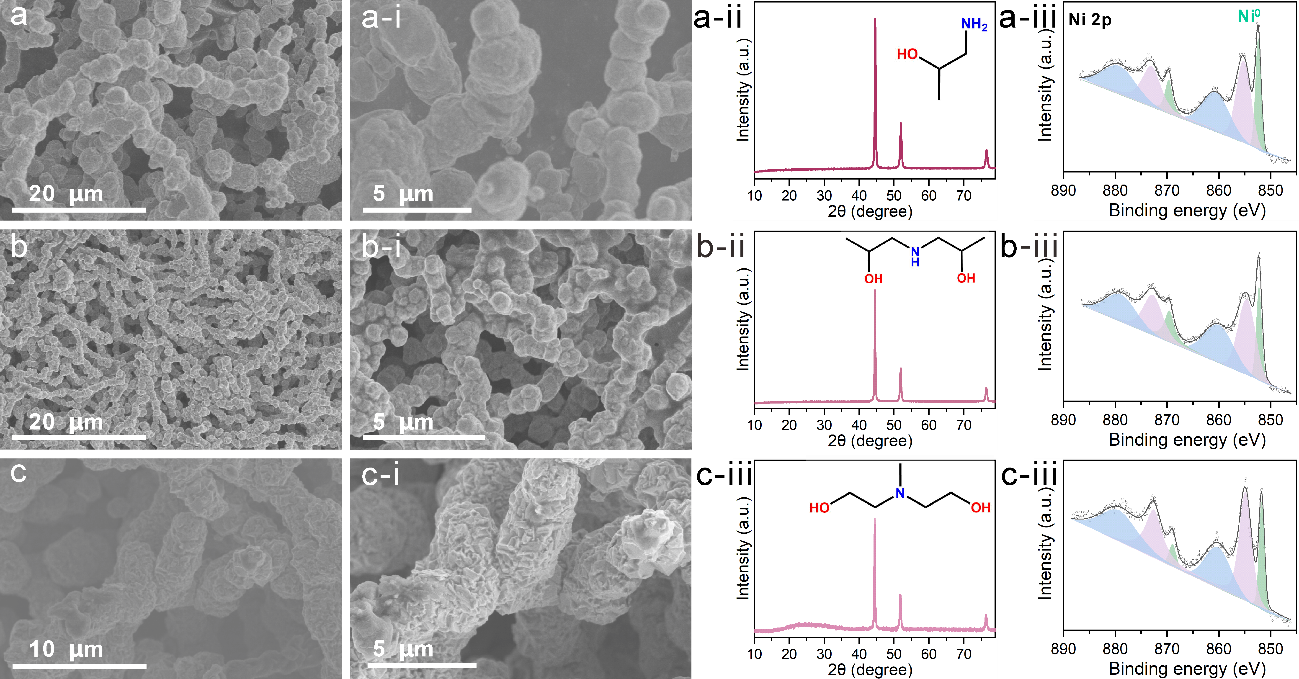


**Figure S3.** The reducing solvent for preparing Ni-based materials is 1-amino-2-propanol (MIPA) (a); diisopropanolamine (DIPA) (b); *N*-methyldiethanolamine (MDEA) (c). (a, a-i; b, b-i; c, c-i) SEM images of the obtained Ni-based materials. (a-ii; b-ii; c-ii) The corresponding XRD patterns. (a-iii; B-iii; C-iii) XPS spectra of theses Ni-based materials.

The as-synthesized Ni-based materials exhibit morphological characteristics analogous to those of Ni-BNSs and Ni-BPHs, featuring interconnected one-dimensional architectures. Furthermore, the XRD diffraction patterns show alignment with the standard pattern of metallic nickel (JCPDS No. 70-0989). These collective observations confirm that the coordination-mediated synthesis employing 1-amino-2-propanol, diisopropanolamine, and *N*-methyldiethanolamine as tailored solvents successfully yields three-dimensional nickel metallic foams with hierarchical porosity.


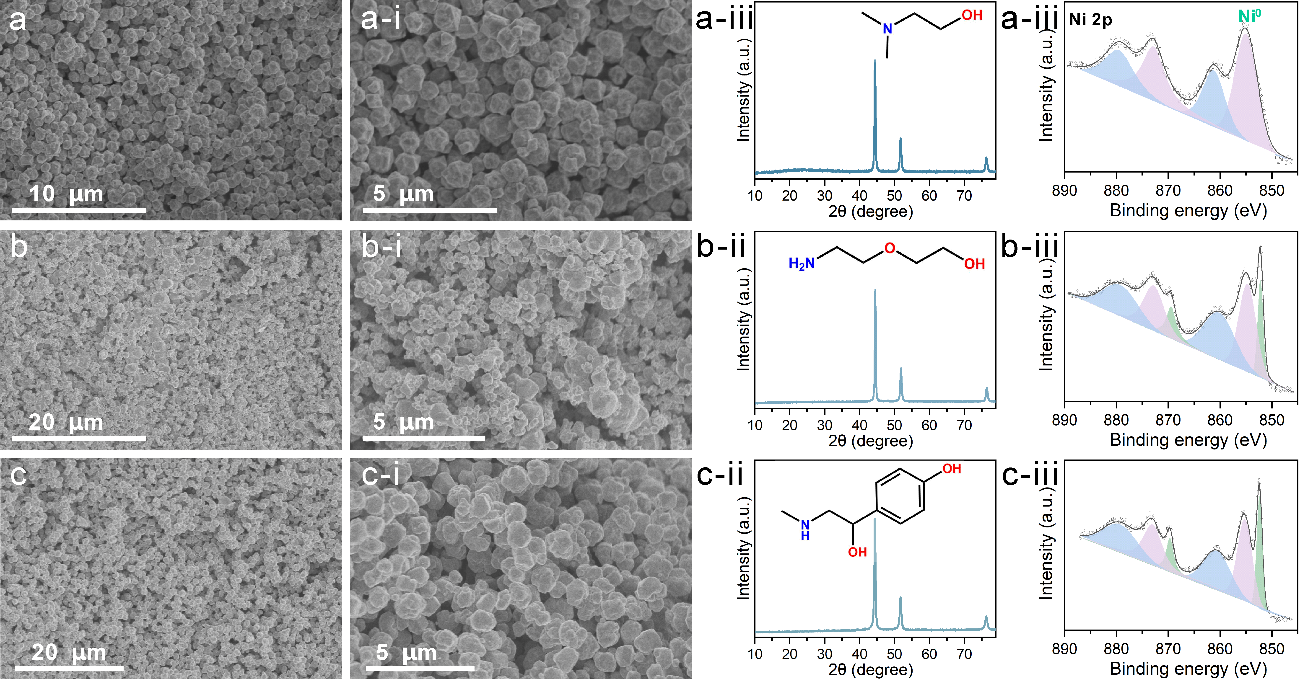


**Figure S4.** The reducing solvent for preparing Ni-based materials is *N*,*N*-dimethylethanolamine (DMEA) (a); diglycolamine (DGA) (b); synephrine (c). (a, a-i; b, b-i; c, c-i) SEM images of the obtained Ni-based materials. (a-ii; b-ii; c-ii) The corresponding XRD patterns. (a-iii; b-iii; c-iii) XPS spectra of theses Ni-based materials.

The as-synthesized Ni-based materials exhibit discrete microspheres. The XRD diffraction patterns show alignment with the standard pattern of metallic nickel (JCPDS No. 70-0989), suggesting that the Ni-based materials prepared with *N*,*N*-dimethylethanolamine, diglycolamine, synephrine as solvents are Ni metal. Nevertheless, these selected solvents exhibit insufficient templating capacity to orchestrate the assembly of one-dimensional architectures.


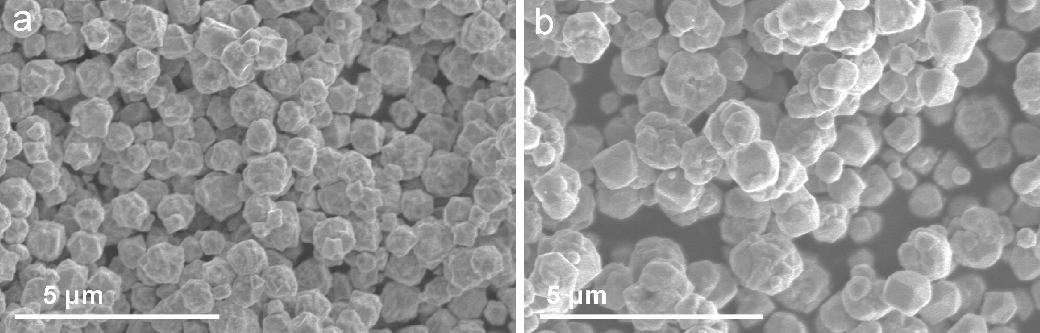


**Figure S5.** The SEM image of the microspheres obtained from the *N*,*N*-dimethylethanolamine system (a) and the image of the product after re-dispersion into the DEA system for reaction (b).

The microspheres initially synthesized in the *N*,*N*-dimethylethanolamine-mediated system were subsequently re-dispersed into a DEA and subjected to secondary hydrothermal treatment at 200 ^o^C for 10 h. SEM image revealed that these nanostructures exhibited remarkable morphological persistence, maintaining their discrete spherical configuration without demonstrating propensity for linear assembly. This observation suggests that the DEA solvent system lacks the necessary anisotropic coordination capability to drive the oriented attachment or directional organization of nickel-based building blocks under the applied thermodynamic conditions.


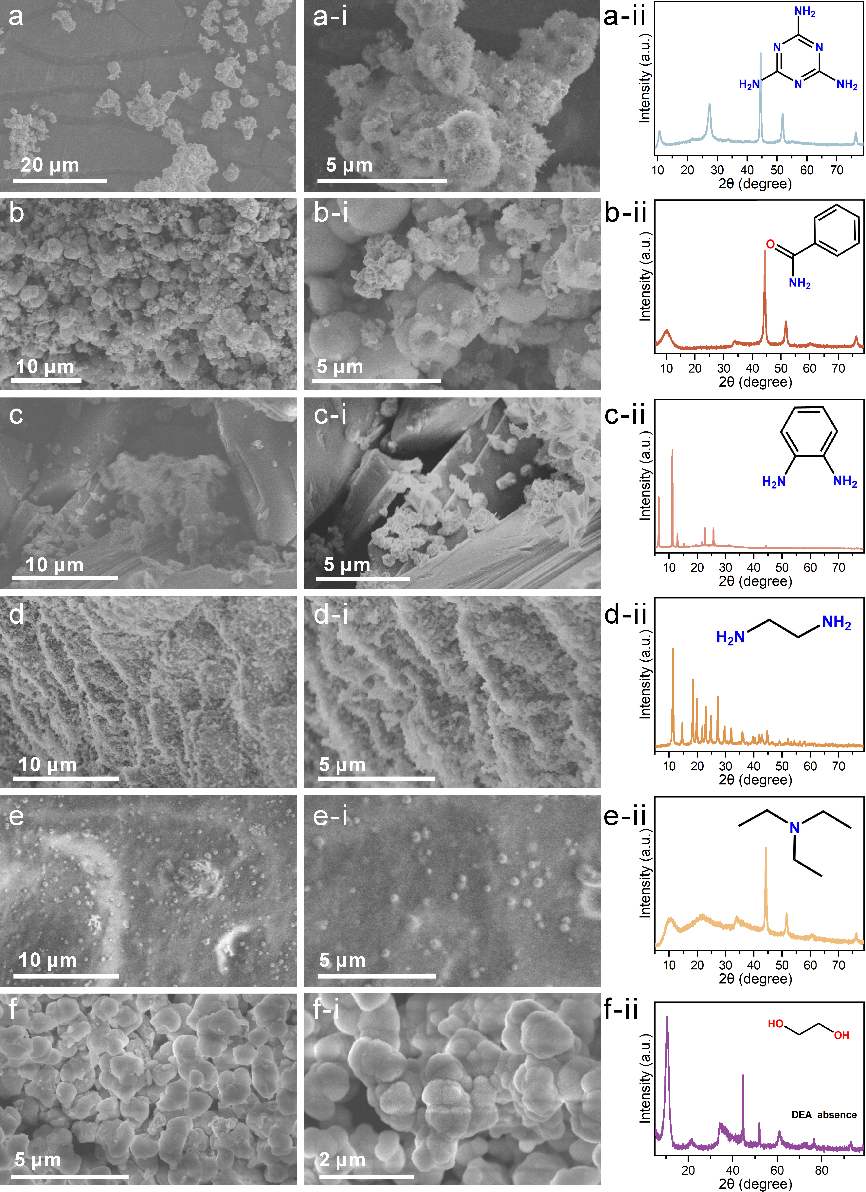


**Figure S6.** The reducing solvent for preparing Ni-based materials is melamine (a); benzamide (b); 1,2-diaminobenzene (c); ethylenediamine (d); triethylamine (e); triethylamine (f). (a, a-i; b, b-i; c, c-i; d, d-i; e, e-i; f, f-i) SEM images of the obtained Ni-based materials. (a-ii; b-ii; c-ii; d-ii; e-ii; f-ii) The corresponding XRD patterns.

The as-synthesized Ni-based materials exhibit undefined morphological features, and their XRD patterns display significant difference compared to the characteristic diffraction signatures of crystalline metallic nickel (JCPDS No. 70-0989). These characteristics indicate that the selected solvent systems fail to induce metallic phase formation.


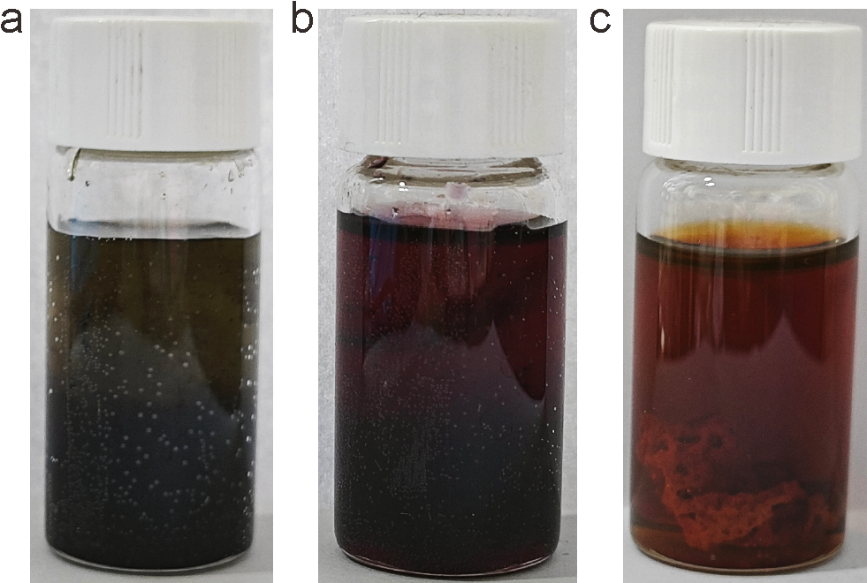


**Figure S7.** Photographs of the solutions after iron ions (a), cobalt ions (b) and copper ions (c) reacted with DEA respectively.


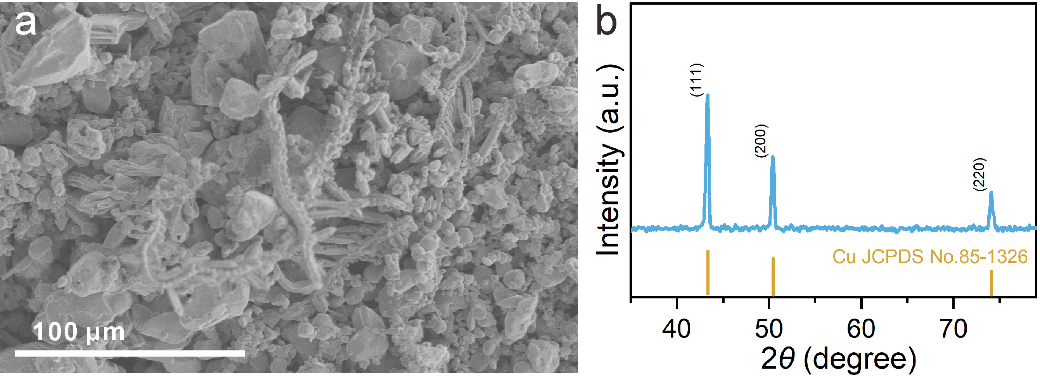


**Figure S8.** (a) SEM image of Cu-based material obtained in the DEA system. (b) Corresponding XRD pattern.


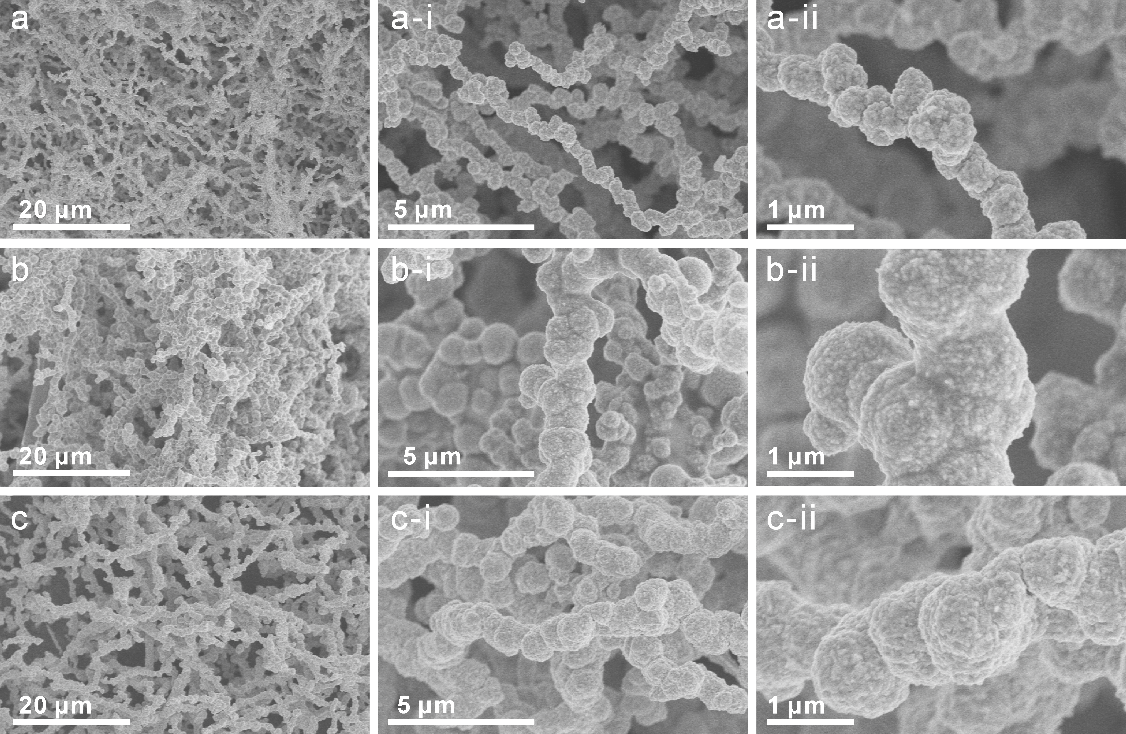


**Figure S9.** SEM images of Ni-BNSs prepared by 0.2 mmol nickel ions (a to a-ii), 0.5 mmol nickel ions (b to b-ii), 2 mmol nickel ions (c to c-ii).

This study reveals that the domain size of metallic nickel can be controlled by simply varying the Ni^2+^ ionic precursor concentration, demonstrating a direct correlation between ionic concentration gradients and the nanoscale dimensional evolution of Ni-BNSs.


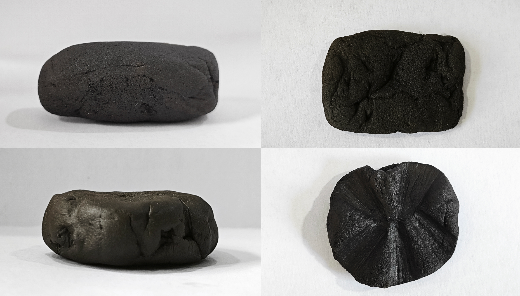


**Figure S10.** Photographs of the Ni-BNSs sponges.


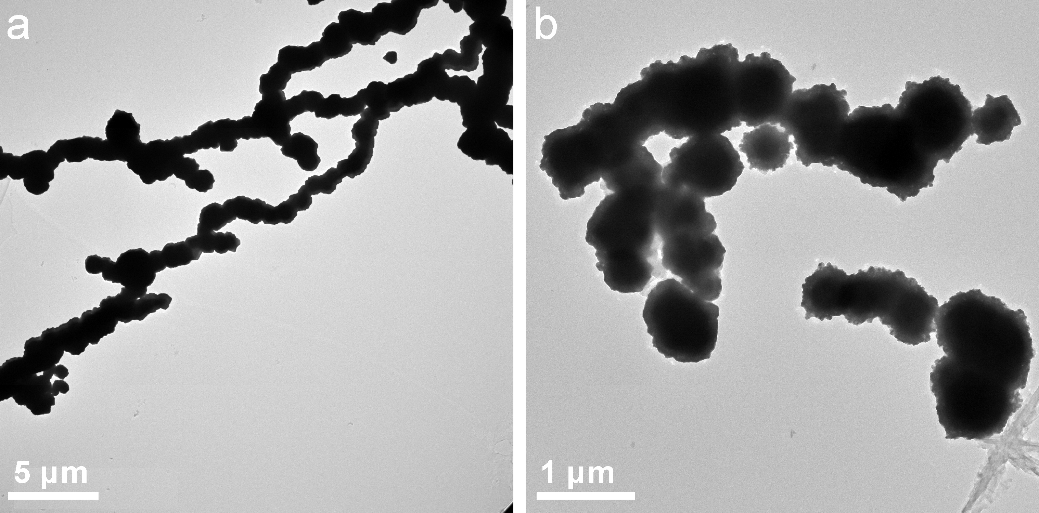


**Figure S11.** TEM images of Ni-BPHs (a) and Ni-BNSs sponges (b).

We engineered Ni-BPHs and Ni-BNSs with a smaller dimension by modulating nickel ion concentrations for TEM characterization.


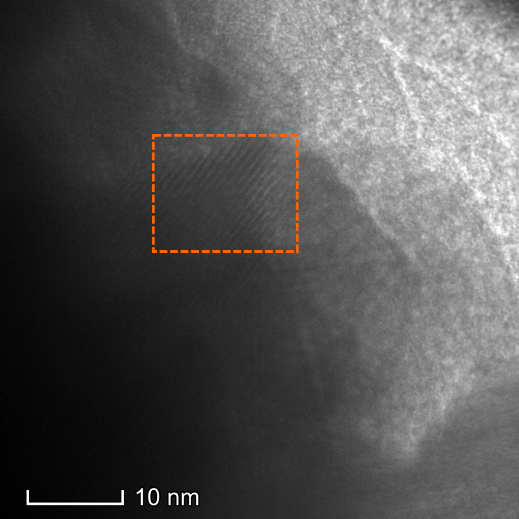


**Figure S12.** HRTEM image of Ni-BNSs sponges.


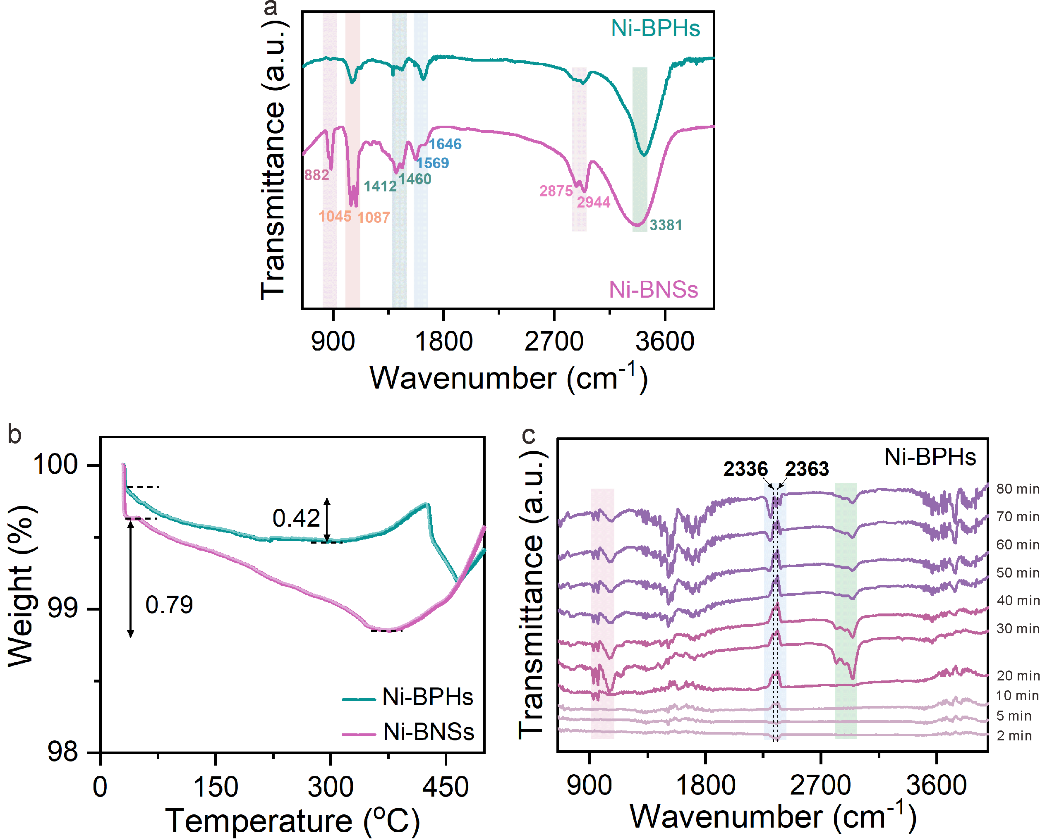


**Figure S13.** (a) FTIR spectra of Ni-BPHs and Ni-BNSs sponges and assignment of main vibrational bands. (b) TGA analyses. (c) FTIR spectra obtained during the in situ thermogravimetric analysis.





**Figure S14.** N 1s XPS spectra of Ni-BPHs and Ni-BNSs sponges.

**

**

**Figure S15.** UV–vis DRS spectra of Ni-BPHs and Ni-BNSs sponges.


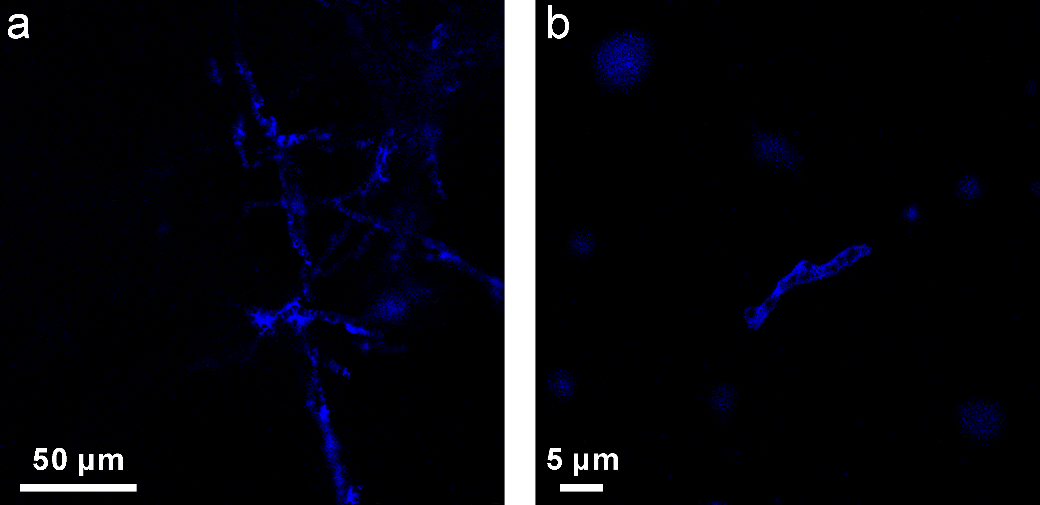


**Figure S16.** The super-resolution fluorescence microscope images of Ni-BPHs (a) and Ni-BNSs sponges (b).


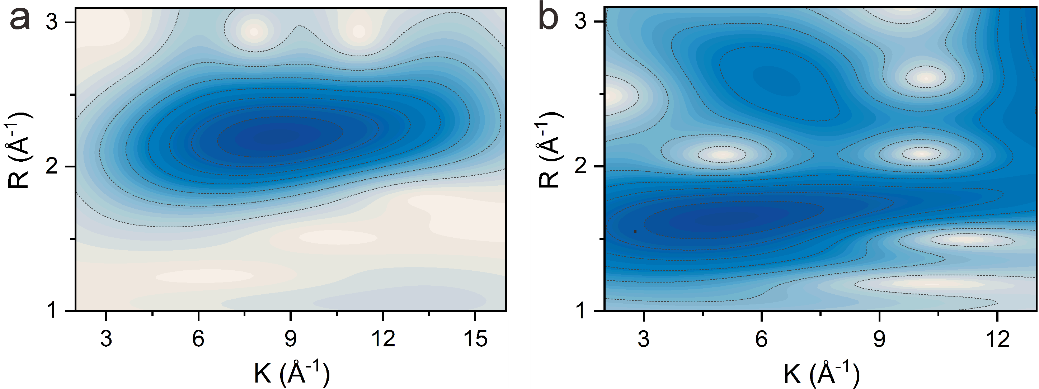


**Figure S17.** The wavelet transform of *k^3^*-weighted EXAFS signals of Ni foil (a) and NiO (b).


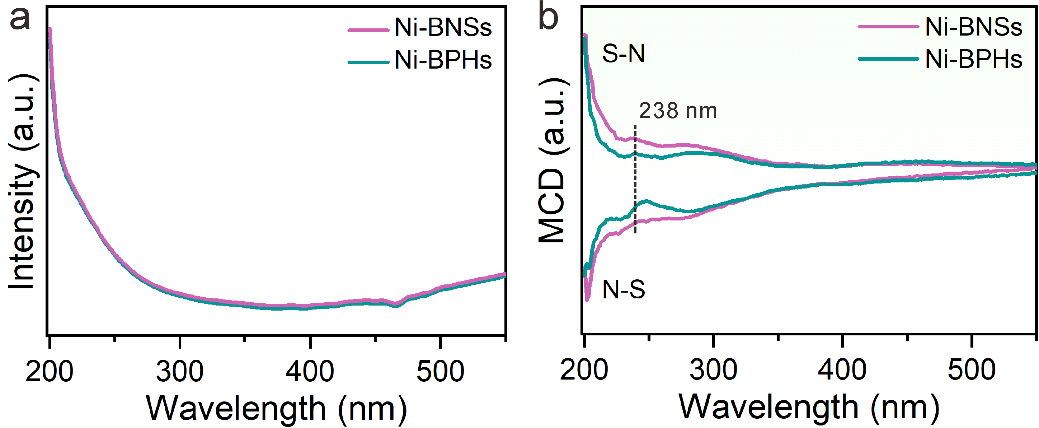


**Figure S18.** UV-vis absorption spectra (a) and magnetic circular dichroism spectroscopy (b) of Ni-BPHs and Ni-BNSs sponges in ethanol.


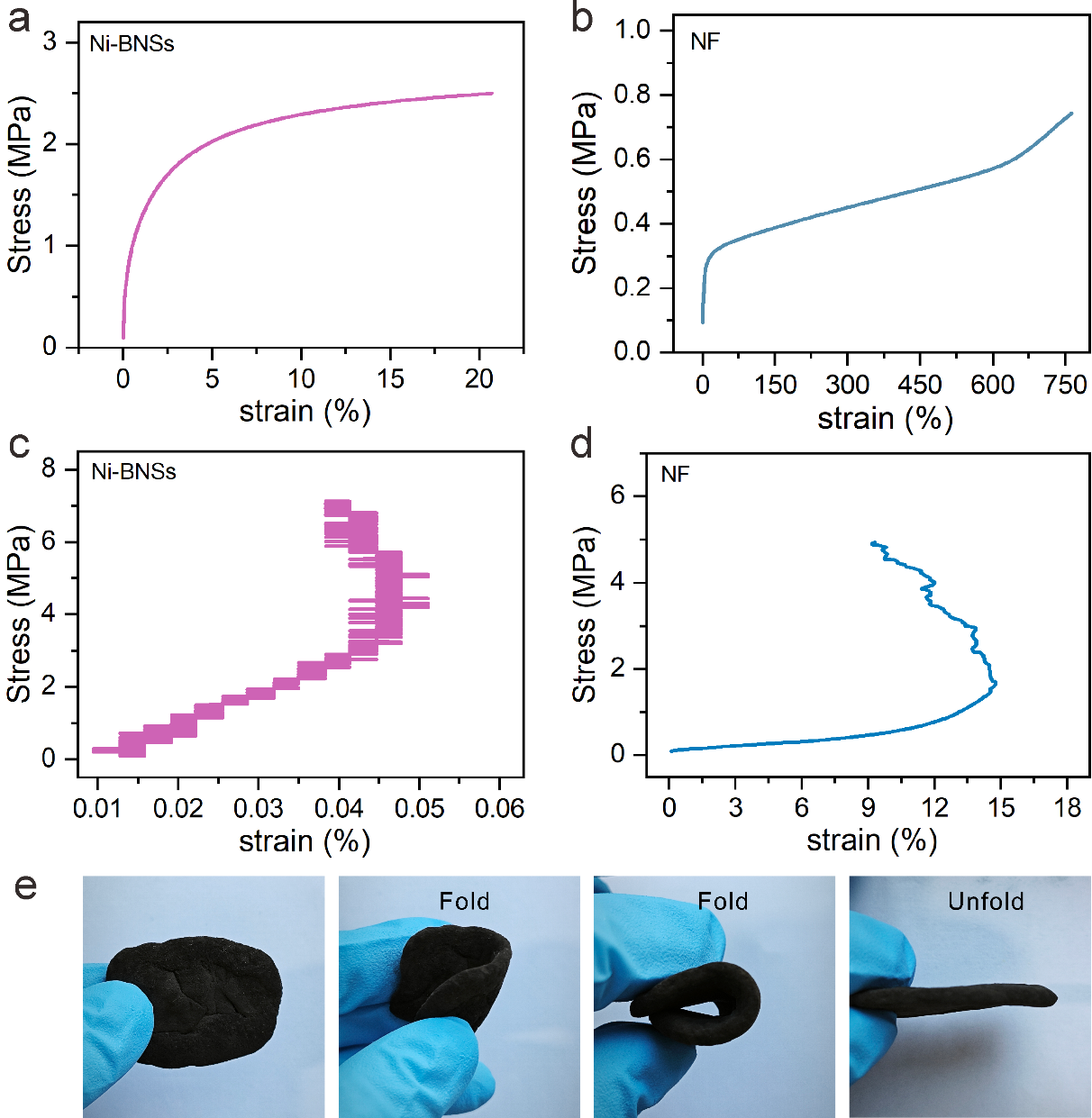


**Figure S19.** Compressive properties of Ni-BNSs (a) and NF (b). Bending properties of Ni-BNSs (c) and NF (d). (e) Optical images demonstrate the superior bendability of Ni-BNSs architectures under extreme bending angles.


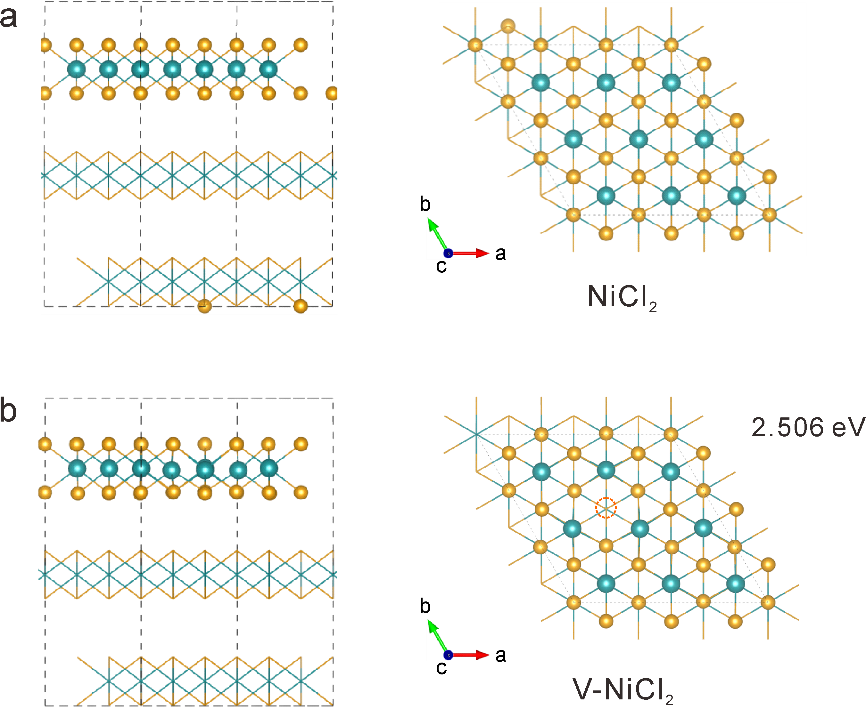


**Figure S20.** Computational models for density functional theory (DFT) simulations. (a) Optimized NiCl_2_ structure. Aqua balls: Ni; Yellow balls: Cl. (b) NiCl_2_ crystal structure with Cl vacancy, showing a calculated vacancy formation energy (2.506 eV).


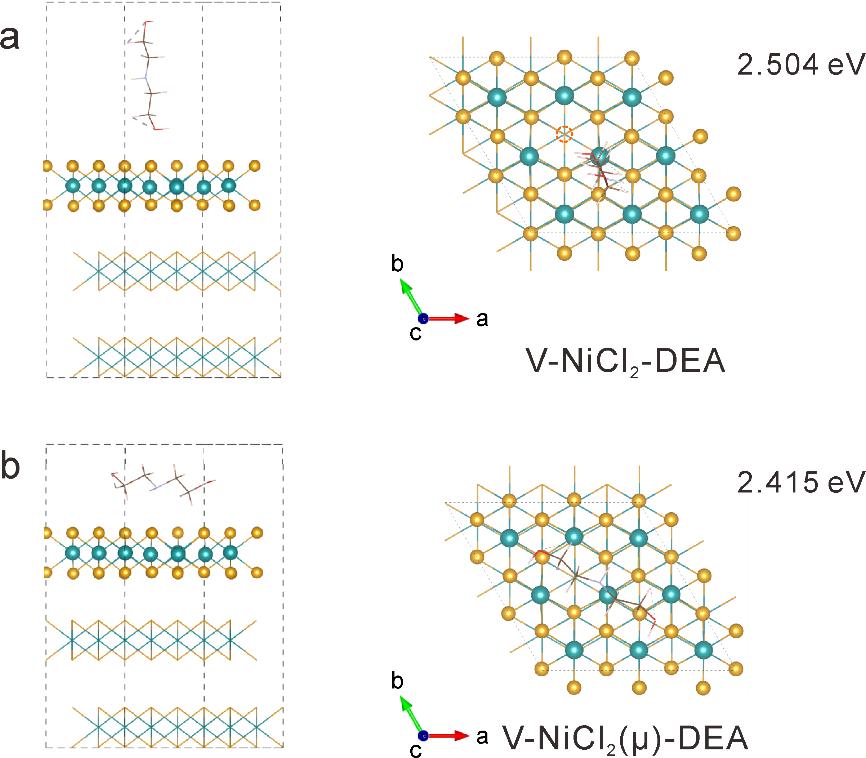


**Figure S21.** (a) DFT-optimized chemisorption of DEA at the Cl vacancy in the surface slab model. The adsorption of DEA onto the NiCl_2_ crystal structure (vacancy generation energy: 2.504 eV). (b) DFT-optimized chemisorption of DEA in the surface slab model with magnetic moment distribution (vacancy generation energy: 2.415 eV).


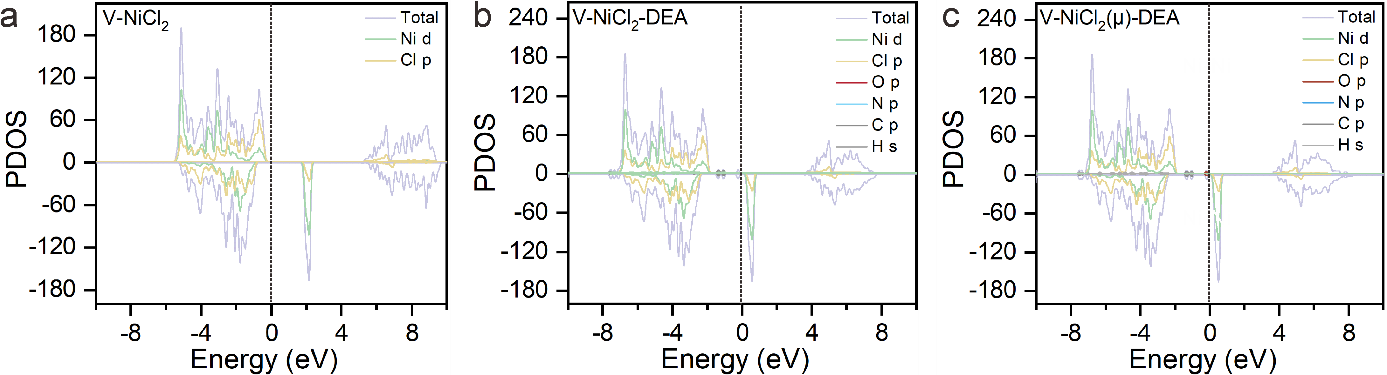


**Figure S22.** Calculated PDOS for the V-NiCl_2_ (a), V-NiCl_2_-DEA (b) and V-NiCl_2_(μ)-DEA (c).


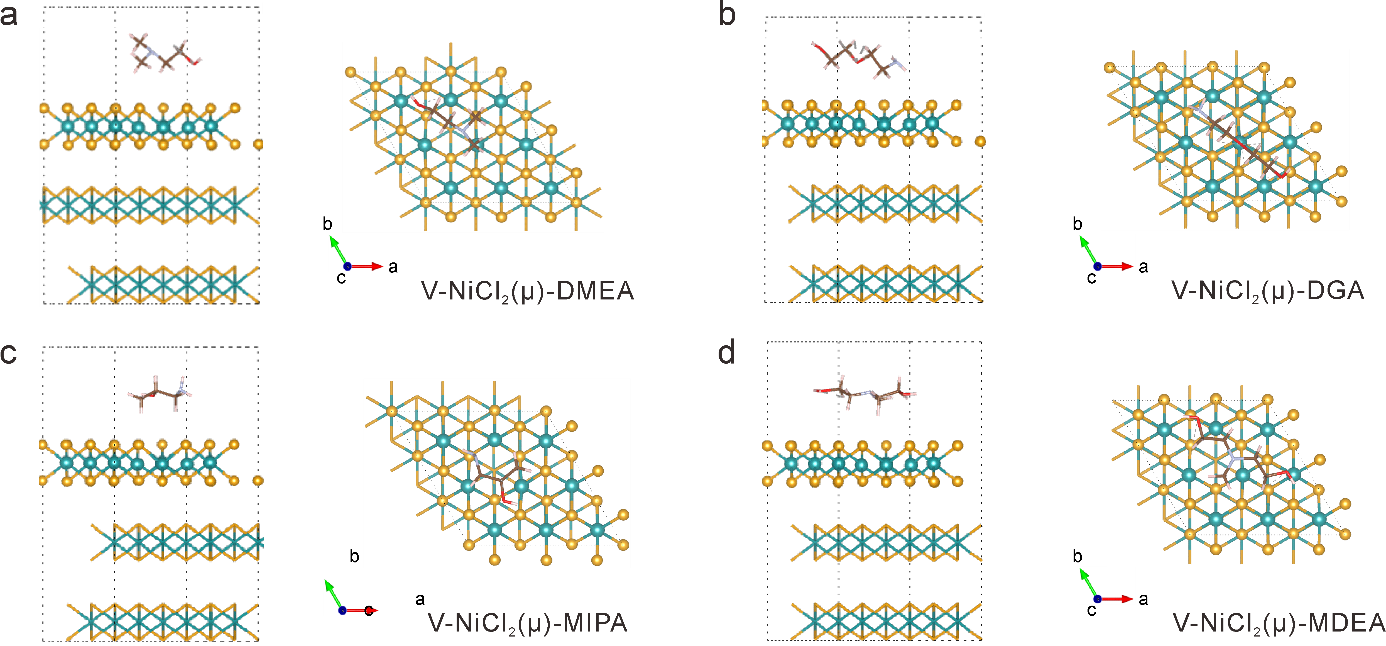


**Figure S23.** The adsorption of DMEA (a), DGA (b), MIPA (c) and MSEA (d) onto the NiCl_2_ crystal structure with Cl vacancy along with magnetic moment distribution.

**
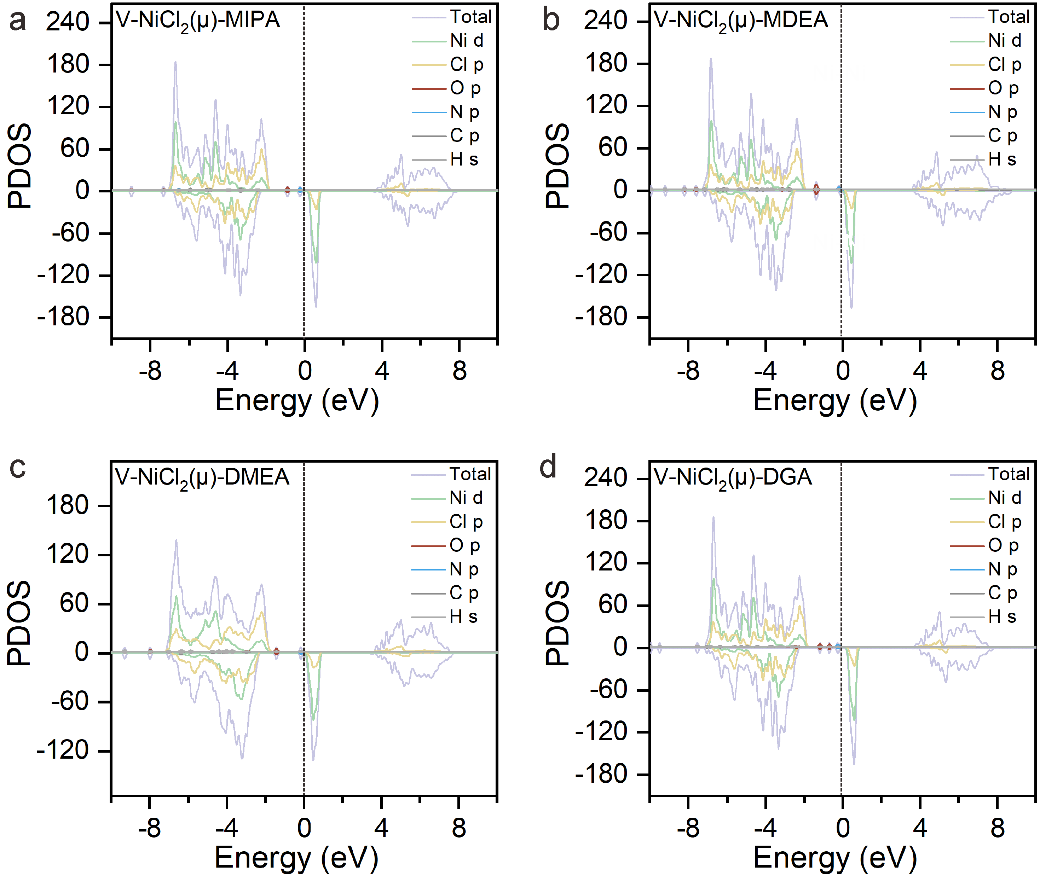
**

**Figure S24.** Calculated PDOS for the V-NiCl_2_(μ)-MIPA (a), V-NiCl_2_(μ)-MDEA (b), V-NiCl_2_(μ)-DMEA (c) and V-NiCl_2_(μ)-DGA (d).

**
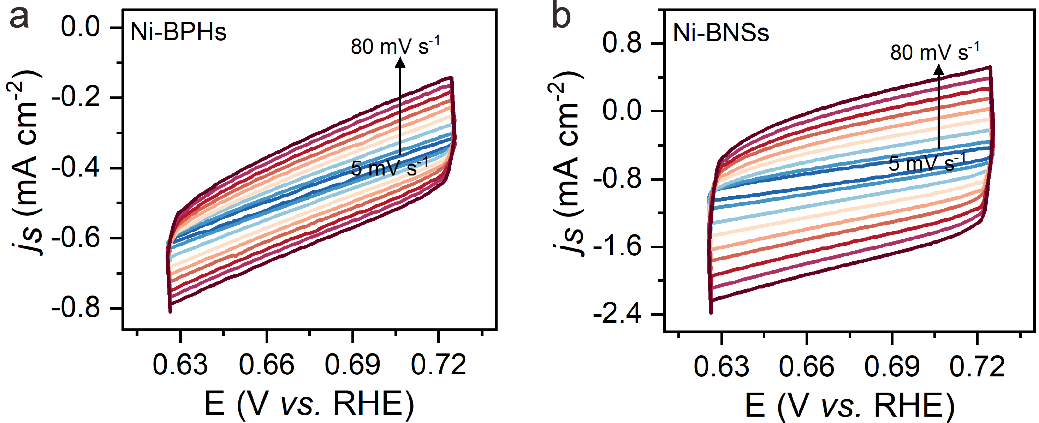
**

**Figure S25.** Double-layer capacitance tests. The cyclic voltammograms (CV) curves of Ni-BPHs (a) and Ni-BNSs sponges (b) at various scan rates of 5, 10, 20, 30, 40, 50, 60, 70, and 80 mV s^-1^ under the potential range of 0.625-0.725 V *vs*. RHE, which were used to estimate double-layer capacitance (C_dl_).

**

**

**Figure S26.** Plots of capacitive currents vs different scan rates with calculated C_dl_.

**

**

**Figure S27.** EIS Nyquist curves of Pt, NF, Ni-BNSs and Ni-BPHs.

**
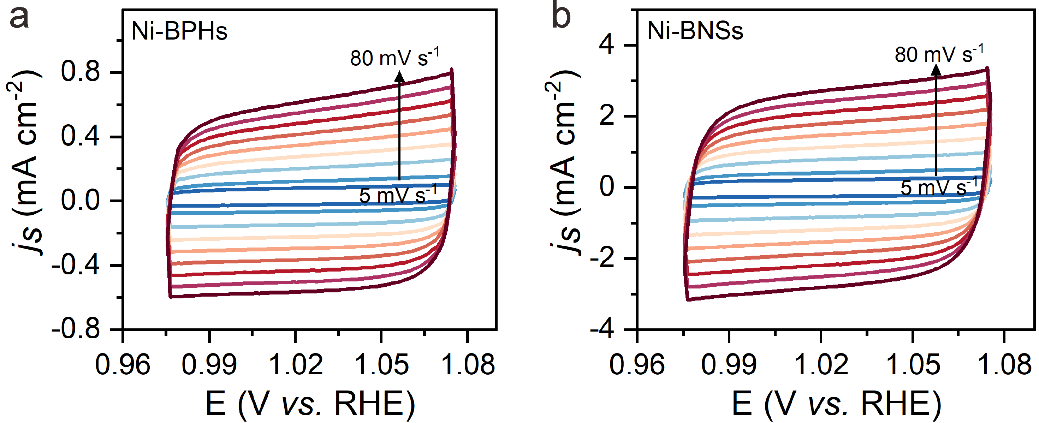
**

**Figure S28.** Double-layer capacitance tests. The CV curves of Ni-BPHs (a) and Ni-BNSs sponges (b) at various scan rates of 5, 10, 20, 30, 40, 50, 60, 70, and 80 mV s^-1^ under the potential range of 0.975-1.075 V *vs*. RHE, which were used to calculate C_dl_ for evaluating the electrochemically active surface area.


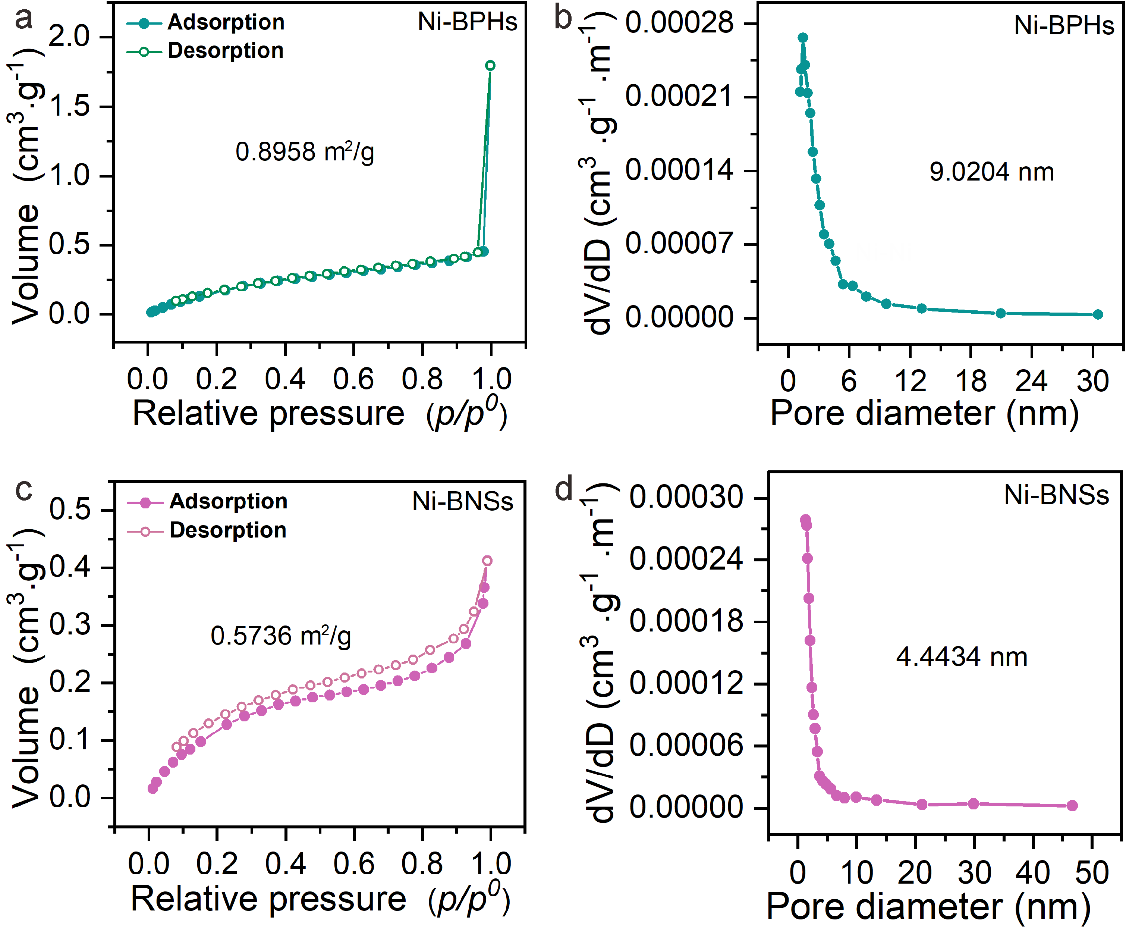


**Figure S29.** N_2_ sorption isotherms of (a) Ni-BPHs and (c) Ni-BNSs, and pore size distribution curves of (b) Ni-BPHs and (d) Ni-BNSs.

Pore volume measurements also reveal exceptionally low values: 0.002020 cm^3^/g for Ni-BPHs and 0.000637 cm^3^/g for Ni-BNSs.

**
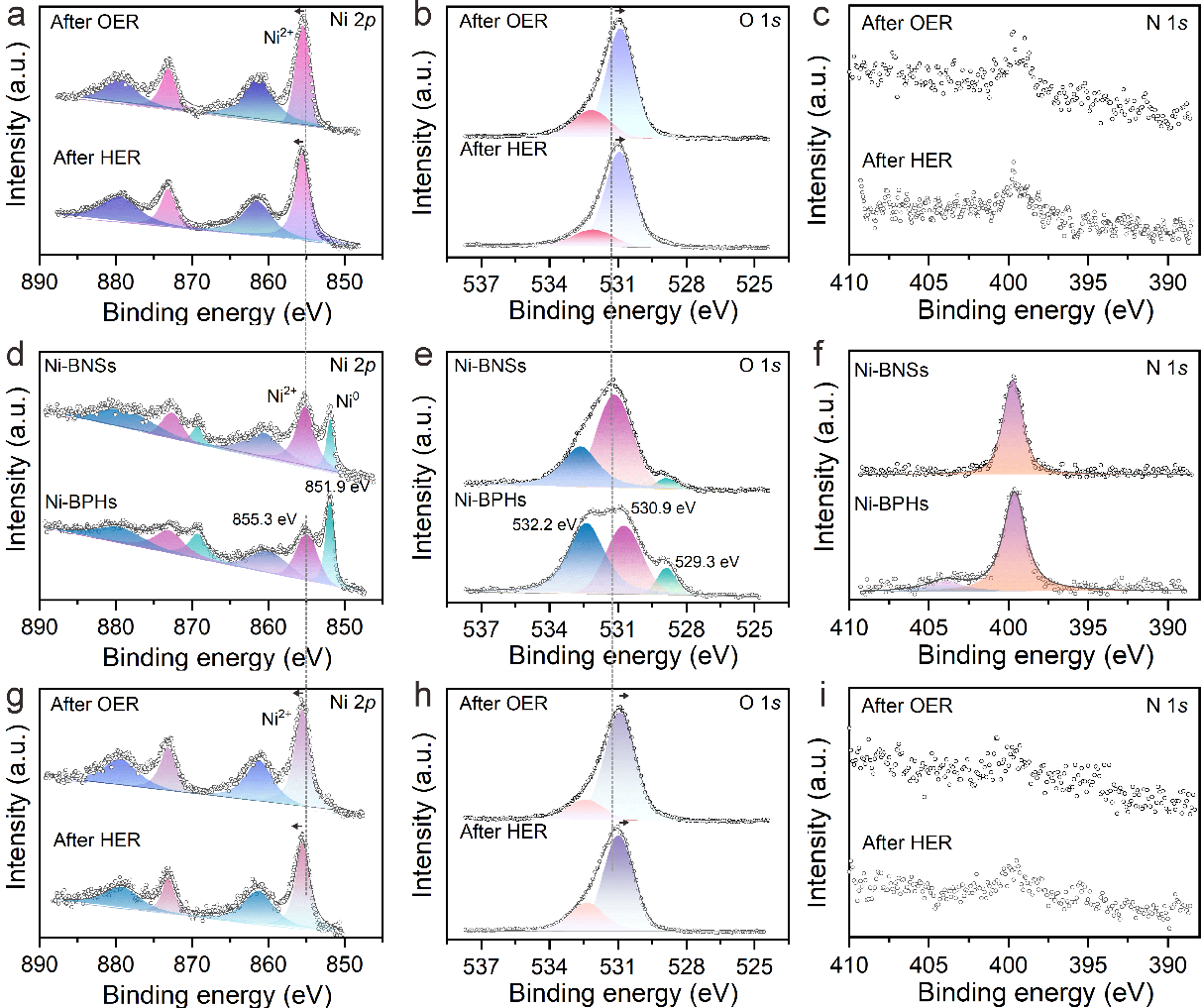
**

**Figure S30.** High-resolution XPS spectra of Ni 2*p* (a), O 1*s* (b) and N 1*s* (c) for Ni-BNSs sponges after HER and OER stability test. XPS spectra of Ni 2*p* (d), O 1*s* (e) and N 1*s* (f) for Ni-BPHs and Ni-BNSs sponges. XPS spectra of Ni 2*p* (g), O 1*s* (h) and N 1*s* (i) for Ni-BPHs sponges after HER and OER stability test.

Post-electrocatalytic analysis via XPS reveals significant electronic restructuring in both Ni-BPH and Ni-BNS systems. The characteristic Ni^0^ peak at 851.9 eV undergoes complete attenuation with a slight shift toward the high binding energy upon HER/OER operation, indicative of surface oxidation elevating the nickel oxidation state to predominant Ni^2+^/Ni^3+^ species. Concurrently, the O 1*s* signal shifts to a little lower binding energy, suggesting the enhanced interaction between Ni and the O. Notably, the complete disappearance of the N 1*s* peak confirms the leaching of ligands after catalytic reactions.


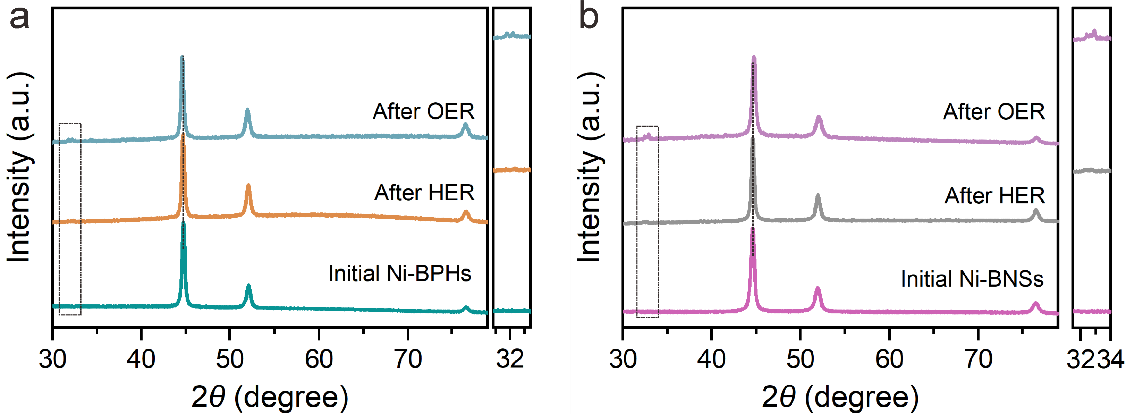


**Figure S31.** XRD patterns of Ni-BPHs (a) and Ni-BNSs sponges (b) before and after HER and OER test.

Post-operando XRD characterization reveals distinct structural evolution pathways under HER and OER conditions. For HER-treated Ni-BPHs/BNSs, the characteristic diffraction of metallic nickel remains prominent, demonstrating remarkable crystallographic stability in reducing environments. In stark contrast, OER-processed specimens emerge reflections at around 32°. This phase transformation confirms potential-driven surface restructuring.


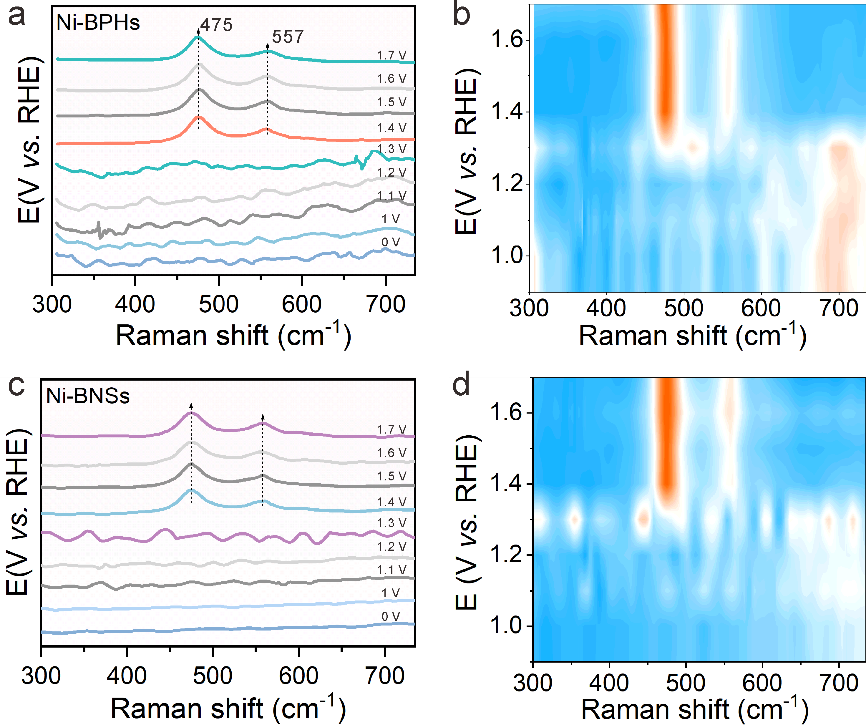


**Figure S32.** Raman spectra and corresponding contour plots of Ni-BPHs (a, b) and Ni-BNSs sponges (c, d) during OER process at different applied potentials (versus reversibly hydrogen electrode (RHE)) in 1.0 M KOH.

**Table S1.** Ni element content in Ni-BPHs and Ni-BNSs was determined by ICP-MS after electrochemical testing.

| **Electrocatalysts** | **Ni (ppb)** | **electrochemical testing** |
| --- | --- | --- |
| Ni-BPHs | 84.8% | after HER |
| Ni-BPHs | 84.4% | after OER |
| Ni-BNSs | 82.6% | after HER |
| Ni-BNSs | 83.9% | after OER |

**Table S2.** Comparison of the electrocatalytic OER performance of Ni metal sponges developed in the current work with that of the previously reported Ni-based electrocatalysts in the literature.

| **Electrocatalysts** | **Electrolyte** | **η (mV vs. RHE)** | **Tafel slope (mV dec^-1^)** | **Ref.** |
| --- | --- | --- | --- | --- |
| Ni-BPHs | 1.0 M KOH | 300 (η_10_) 325 (η_20_) | 18 | This work |
| Ni-BNSs | 1.0 M KOH | 290 (η_10_) 313 (η_20_) | 17 | This work |
| NiSA-O/Mo_2_C | 1.0 M KOH | 299 (η_10_) | 89.36 | ^[4]^ |
| MoS_2_/Ni_3_S_2_/NF | 1.0 M KOH | 239 (η_10_) | 71 | ^[5]^ |
| FeP/Fe_3_C@NPC | 1.0 M KOH | 440 (η_10_) | 108.6 | ^[6]^ |
| NiMoO_x_/NiMoS | 1.0 M KOH | 186 (η_10_) | 34 | ^[7]^ |
| Ni_3_S_2_/NF | 1.0 M NaOH | 260 (η_10_) |  | ^[8]^ |
| Fe-Co_2_P@Fe-N-C | 1.0 M KOH | 300 (η_10_) | 79 | ^[9]^ |
| FeNi/NPC | 0.1 M KOH | 310 (η_10_) | 62 | ^[10]^ |
| N-NiMoO_4_/Ni/CNTs | 1.0 M KOH | 330 (η_10_) | 89.5 | ^[11]^ |
| Mo-FeNiP NTs/NF | 1.0 M KOH | 182.5 (η_10_) | 64.7 | ^[12]^ |
| V_0.3_-NiS/NiS_2_ | 1.0 M KOH | 220 (η_10_) | 72 | ^[13]^ |
| Ru–NiSe_2_/NF | 1.0 M KOH | 210 (η_10_) | 60.5 | ^[14]^ |

**Table S3.** Comparison of the electrocatalytic HER performance of Ni metal sponges developed in the current work with that of the previously reported Ni-based electrocatalysts in the literature.

| **Electrocatalysts** | **Electrolyte** | **η (mV vs. RHE)** | **Tafel slope (mV dec^-1^)** | **Ref.** |
| --- | --- | --- | --- | --- |
| Ni-BPHs | 1.0 M KOH | 50 (η_10_) 88 (η_20_) | 136 | This work |
| Ni-BNSs | 1.0 M KOH | 31 (η_10_) 56 (η_20_) | 139 | This work |
| NiSA-O/Mo_2_C | 1.0 M KOH | 133 (η_10_) | 83.6 | ^[4]^ |
| MoS_2_/Ni_3_S_2_/NF | 1.0 M KOH | 203 (η_10_) | 98 | ^[5]^ |
| FeP/Fe_3_C@NPC | 1.0 M KOH | 97 (η_10_) | 89.3 | ^[6]^ |
| NiMoO_x_/NiMoS | 1.0 M KOH | 38 (η_10_) | 38 | ^[7]^ |
| Ni_3_S_2_/NF | 1.0 M NaOH | 223 (η_10_) |  | ^[8]^ |
| Fe-Co_2_P@Fe-N-C | 1.0 M KOH | 77 (η_10_) | 56 | ^[9]^ |
| FeNi/NPC | 0.1 M KOH | 260 (η_10_) | 112 | ^[10]^ |
| N-NiMoO_4_/Ni/CNTs | 1.0 M KOH | 48 (η_10_) | 58.1 | ^[11]^ |
| Mo-FeNiP NTs/NF | 1.0 M KOH | 30 (η_10_) | 76.2 | ^[12]^ |
| V_0.3_-NiS/NiS_2_ | 1.0 M KOH | 94 (η_10_) | 82 | ^[13]^ |
| Ru–NiSe_2_/NF | 1.0 M KOH | 59 (η_10_) | 72.2 | ^[14]^ |

References

[1] a) G. Kresse, J. Furthmüller, *Comput. Mater. Sci.* **1996**, *6*, 15-50; b) G. Kresse, J. Furthmüller, *Phys. Rev. B*. **1996**, *54*, 11169-11186.

[2] a) P. E. Blöchl, *Phys. Rev. B*. **1994**, *50*, 17953-17979; b) G. Kresse, D. Joubert, *Phys. Rev. B*. **1999**, *59*, 1758-1775.

[3] B. Hammer, L. B. Hansen, J. K. Nørskov, *Phys. Rev. B*. **1999**, *59*, 7413-7421.

[4] M. Hou, L. Zheng, D. Zhao, X. Tan, W. Feng, J. Fu, T. Wei, M. Cao, J. Zhang, C. Chen, *Nat. Commun.* **2024**, *15*, 1342.

[5] Y. Yang, H. Yao, Z. Yu, S. M. Islam, H. He, M. Yuan, Y. Yue, K. Xu, W. Hao, G. Sun, H. Li, S. Ma, P. Zapol, M. G. Kanatzidis, *J. Am. Chem. Soc.* **2019**, *141*, 10417-10430.

[6] Y. Li, G. Tang, Y. Wang, Y. Chai, C. Liu, *ACS Omega*. **2022**, *7*, 13687-13696.

[7] P. Zhai, Y. Zhang, Y. Wu, J. Gao, B. Zhang, S. Cao, Y. Zhang, Z. Li, L. Sun, J. Hou, *Nat. Commun.* **2020**, *11*, 5462.

[8] L.-L. Feng, G. Yu, Y. Wu, G.-D. Li, H. Li, Y. Sun, T. Asefa, W. Chen, X. Zou, *J. Am. Chem. Soc.* **2015**, *137*, 14023-14026.

[9] X.-W. Lv, W.-S. Xu, W.-W. Tian, H.-Y. Wang, Z.-Y. Yuan, *Small*. **2021**, *17*, 2101856.

[10] H.-X. Zhong, J. Wang, Q. Zhang, F. Meng, D. Bao, T. Liu, X.-Y. Yang, Z.-W. Chang, J.-M. Yan, X.-B. Zhang, *Adv. Sustainable Syst.* **2017**, *1*, 1700020.

[11] G.-L. Li, X.-Y. Qiao, Y.-Y. Miao, T.-Y. Wang, F. Deng, *Small*. **2023**, *19*, 2207196.

[12] X. Wang, J. Zhou, W. Cui, F. Gao, Y. Gao, F. Qi, Y. Liu, X. Yang, K. Wang, Z. Li, Y. Yang, J. Chen, W. Sun, L. Sun, H. Pan, *Adv. Sci.* **2024**, *11*, 2401207.

[13] W. Xu, R. Zhao, Q. Li, B. Sun, J. Wu, W. Zhong, Y. Gao, X. Nan, Q. Huang, Y. Yang, X. Li, N. Yang, Q. Zhang, *Adv. Energy Mater.* **2023**, *13*, 2300978.

[14] R. Qin, P. Wang, Z. Li, J. Zhu, F. Cao, H. Xu, Q. Ma, J. Zhang, J. Yu, S. Mu, *Small*. **2022**, *18*, 2105305.
